# Immune modulating nanoparticles for the treatment of ocular diseases

**DOI:** 10.1186/s12951-022-01658-5

**Published:** 2022-11-24

**Authors:** Lianfei Fang, Jin Liu, Zeyang Liu, Huifang Zhou

**Affiliations:** 1 Department of Ophthalmology, Ninth People’s Hospital, Shanghai JiaoTong University School of Medicine, Shanghai, China; 2 Shanghai Key Laboratory of Orbital Diseases and Ocular Oncology, 200011 Shanghai, China

**Keywords:** Nanoparticles, Ocular diseases, Anti-inflammatory mechanisms, Treatment

## Abstract

Ocular diseases are increasingly influencing people’s quality of life. Complicated inflammatory mechanisms involved in the pathogenic process of ocular diseases make inflammation-targeting treatment a potential therapeutic approach. The limited efficacy of conventional anti-inflammatory therapeutic strategies, caused by various objective factors, such as complex ocular biological barriers, and subjective factors, such as poor compliance, are promoting the development of new therapeutic methods. With the advantages of considerable tissue permeability, a controllable drug release rate, and selective tissue targeting ability, nanoparticles have successfully captured researchers’ attention and have become a research hotspot in treating ocular diseases. This review will focus on the advantages of nanosystems over traditional therapy, the anti-inflammation mechanisms of nanoparticles, and the anti-inflammatory applications of nanoparticles in different ocular diseases (ocular surface diseases, vitreoretinopathy, uveal diseases, glaucoma, and visual pathway diseases). Furthermore, by analyzing the current situation of nanotherapy and the challenges encountered, we hope to inspire new ideas and incentives for designing nanoparticles more consistent with human physiological characteristics to make progress based on conventional treatments. Overall, some progress has been made in nanoparticles for the treatment of ocular diseases, and nanoparticles have rather broad future clinical translation prospects.

**Figure:**
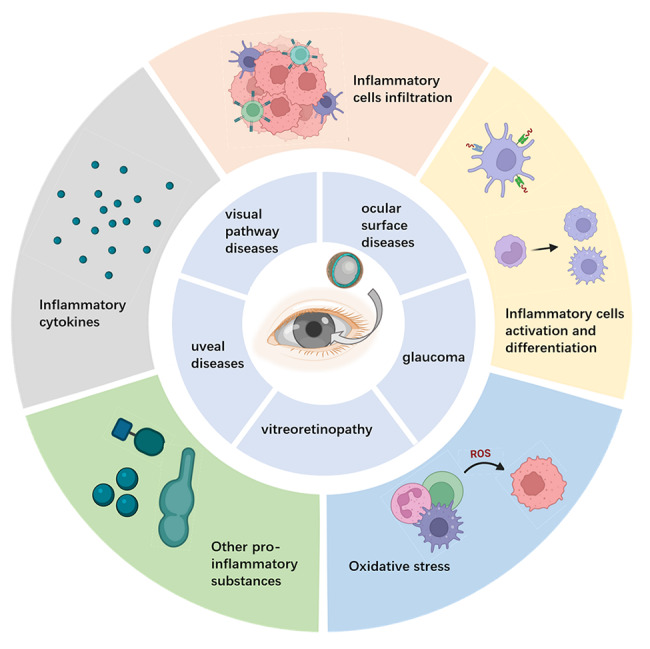
Graphical abstract

Graphical abstract

## Introduction

Ocular diseases significantly influence the quality of life of humans. According to a survey covering 39 countries, 285 million people suffer from visual impairment or blindness, especially elderly individuals[1]. Furthermore, some researchers predict that numbers will double between 2015 and 2050[2]. Inflammation has been demonstrated to be involved in the pathogenesis of many ocular diseases and can be induced by a variety of pathogenic processes, such as the excessive release of inflammatory cytokines, abnormal activation of inflammatory cells, and excessive oxidative stress[3, 4]. An upregulation of proinflammatory cytokines, chemokines and other proinflammatory substances and decreased levels of anti-inflammatory cytokines can be detected in the disease state. Several inflammatory pathways, including the toll-like receptor (TLR) 4, nuclear factor kappa-B (NF-κB), intercellular adhesion molecule-1 (ICAM-1), c-Jun N-terminal kinase (JNK) and P38 mitogen-activated protein kinase (MAPK) pathways, as well as the hypoxia-inducible factor (HIF)-1α-vascular endothelial growth factor (VEGF) pathway, are also involved in contributing to these pathological changes. All these pathological processes can be regarded as potential therapeutic targets for ocular diseases.

Regular anti-inflammatory drugs, such as dexamethasone and triamcinolone acetonide (TA), are currently widely used as anti-inflammatory and antibacterial agents for ocular inflammatory diseases. However, objective factors (such as the existence of ocular physiological barriers and the low solubility of drugs in physiologically compatible solvents) and subjective factors (such as low patient compliance caused by long-term treatment cycles and frequent injections to maintain adequate drug concentrations after rapid drug clearance) limit the efficacy and practicality of these drugs[5, 6]. Some emerging biological agents, such as anti-VEGF drugs, have similar problems. Represented by ralizumab, the rapid degradation rates lead to the need for repeated injections, and the strong hydrophilicity and high molecular weight of these biological agents weaken their ability to pass through tissue barriers and cell membranes[3, 7, 8]. In addition to the imperfect properties of existing drugs, there are many shortcomings in terms of administration routes. Conventional eye drops are often degraded by tears and blinking, which causes much drug wastage, with less than 1–5% of the doses of these drops being delivered to the target tissue[9–12]. Intravitreal injections run the risk of causing pain, intraocular infections and even partial retinal detachment[13, 14]. Hence, novel materials and drug delivery routes need to be developed to replace traditional therapeutic strategies for better treatment effects.

The incorporation of nanomaterials into the intervention process is an excellent idea due to the unique properties of nanomaterials, such as small and controllable particle size, uniform dispersion, diverse selectivity of materials, specific surface structures and a variety of modifiers[15, 16]. The earliest application of nanoparticles (NPs) in the treatment of ocular diseases can be traced back to 1986. T Harmia et al. encapsulated rutin into NPs for ocular delivery[17]. Subsequently, experiments on NPs for the treatment of ocular diseases through inflammatory mechanisms began to be widely carried out in 2002. These experiments demonstrated the anti-inflammatory properties of NPs and confirmed that intravitreal injections did not cause autoimmune-induced visual impairment[18, 19]. An increasing number of NP types have been applied in experiments to treat ocular diseases through anti-inflammatory mechanisms.

**Fig. 1:**
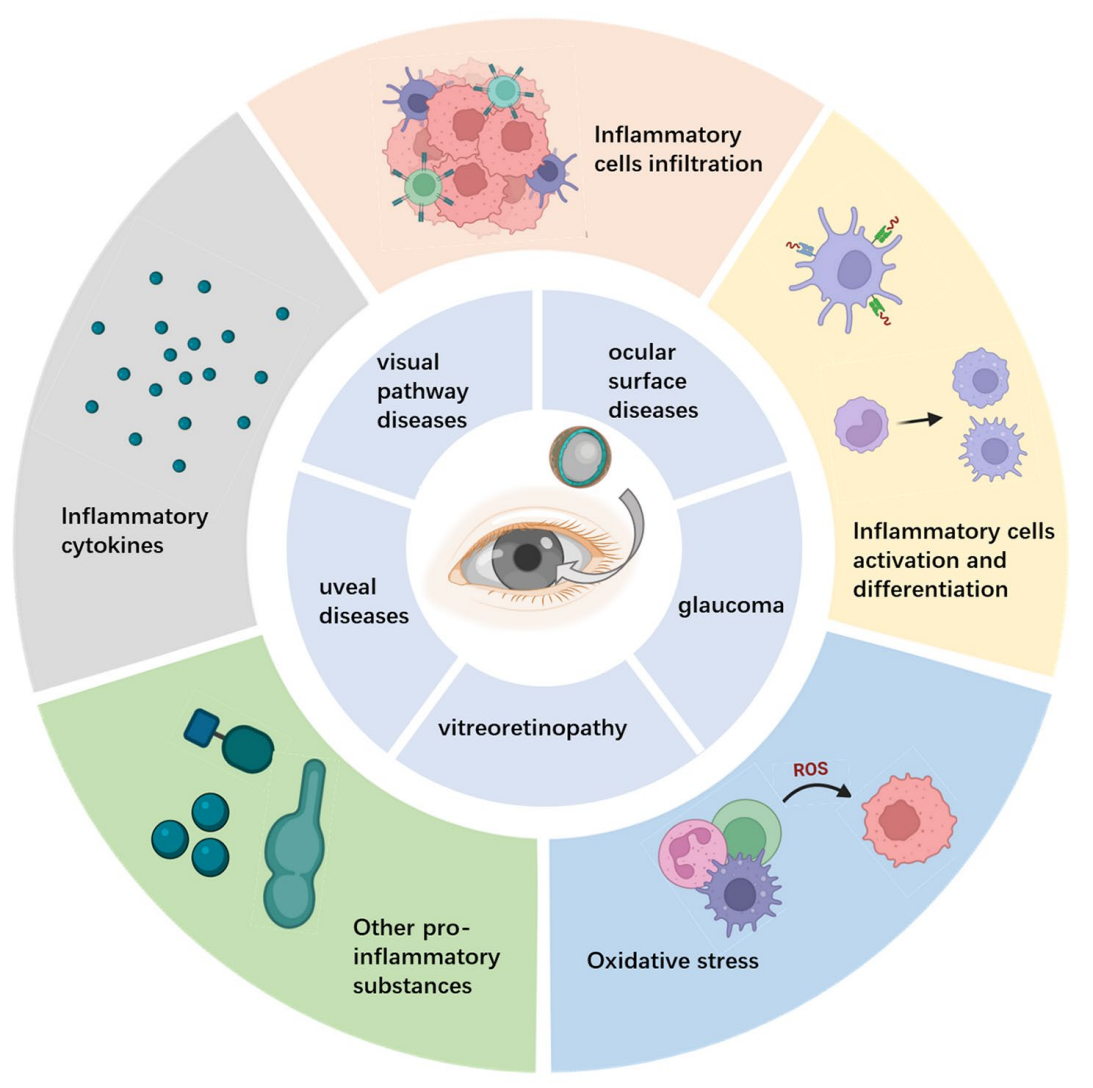
Anti-inflammatory mechanisms of nanoparticles in treating diverse ocular diseases.

Here, by summarizing the findings in articles published in the past five years, we highlight several advantages of NPs to explain why NPs have been selected as potential alternatives to traditional therapies, such as improving drug bioavailability, prolonging the duration of drug effectivity, and improving the drug targeting ability. Then, we summarize several types of anti-inflammatory mechanisms of NPs in treating ocular diseases, including modulating cytokine production and secretion, modulating immune cell activation and differentiation, inhibiting immune cell infiltration, alleviating oxidative stress, and blocking the excretion of other inflammation-related molecules. Ocular diseases in this review are mainly classified into three categories, ocular surface diseases, vitreoretinopathy, and other ocular diseases (uveal diseases, glaucoma, and visual pathway diseases), to introduce their anti-inflammatory mechanisms, as illustrated in Fig. 1. For each ocular disease, we describe the inflammation-related pathogenic process and summarize the anti-inflammatory applications of NPs. In addition to the advantages of NPs mentioned earlier, we analyze the current clinical translation status of NP strategies and challenges faced during research, including the unclear relationship between the physical and chemical properties of NPs and their functions, difficulties of clinical translation due to differences in animal and human ocular anatomy, risks of inflammation after intraocular administration, etc. Despite these challenges, as new materials, nanomaterials can undeniably significantly influence the field of ocular disease treatment.

**Fig. 2:**
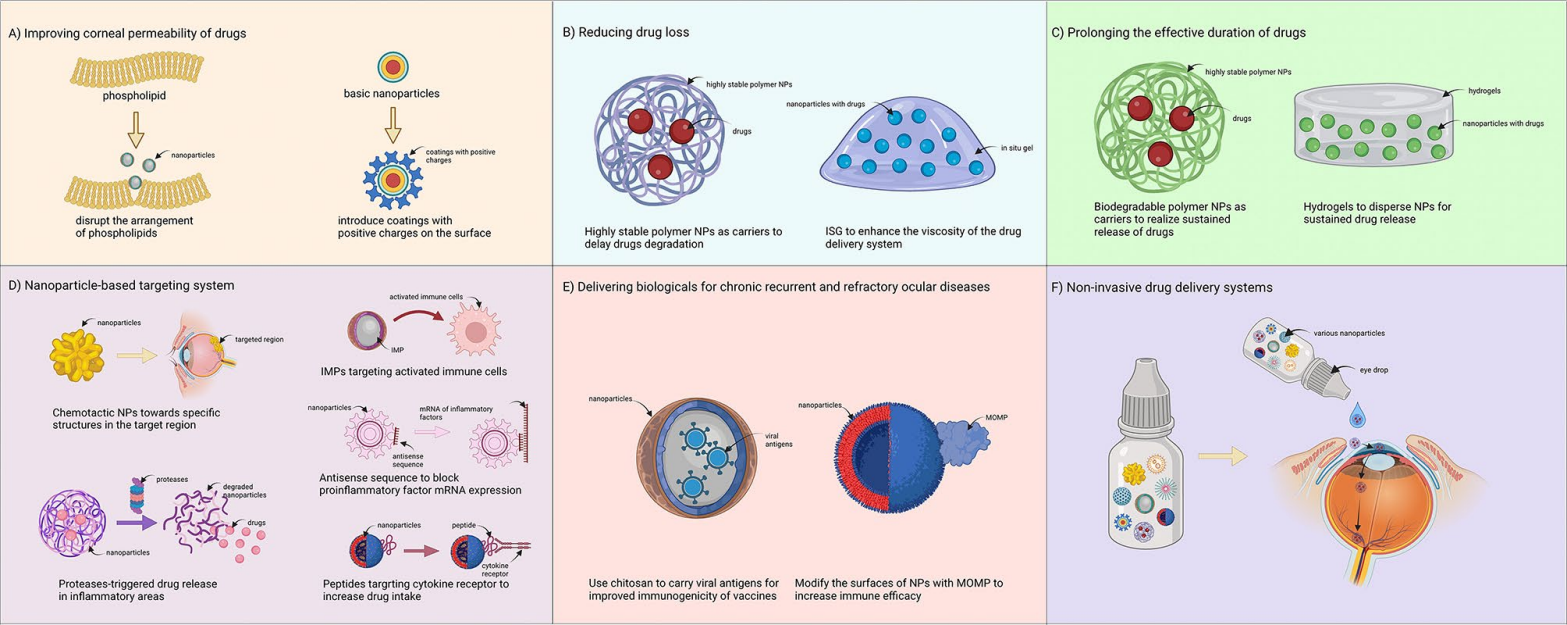
Advantages of nanoparticles over conventional therapies.

## Advantages of nanoparticles over conventional **therapies**

The properties of NPs, such as their small and controllable particle size and the diverse selectivity of nanomaterials, allow the application of NPs based on conventional therapeutic methods in treating ocular diseases. Here, we summarize several advantages of NPs for ocular disease treatment, including improving drug corneal permeability, prolonging the drug effective duration, and achieving targeted drug delivery, as illustrated in Fig. 2.

**Fig. 3:**
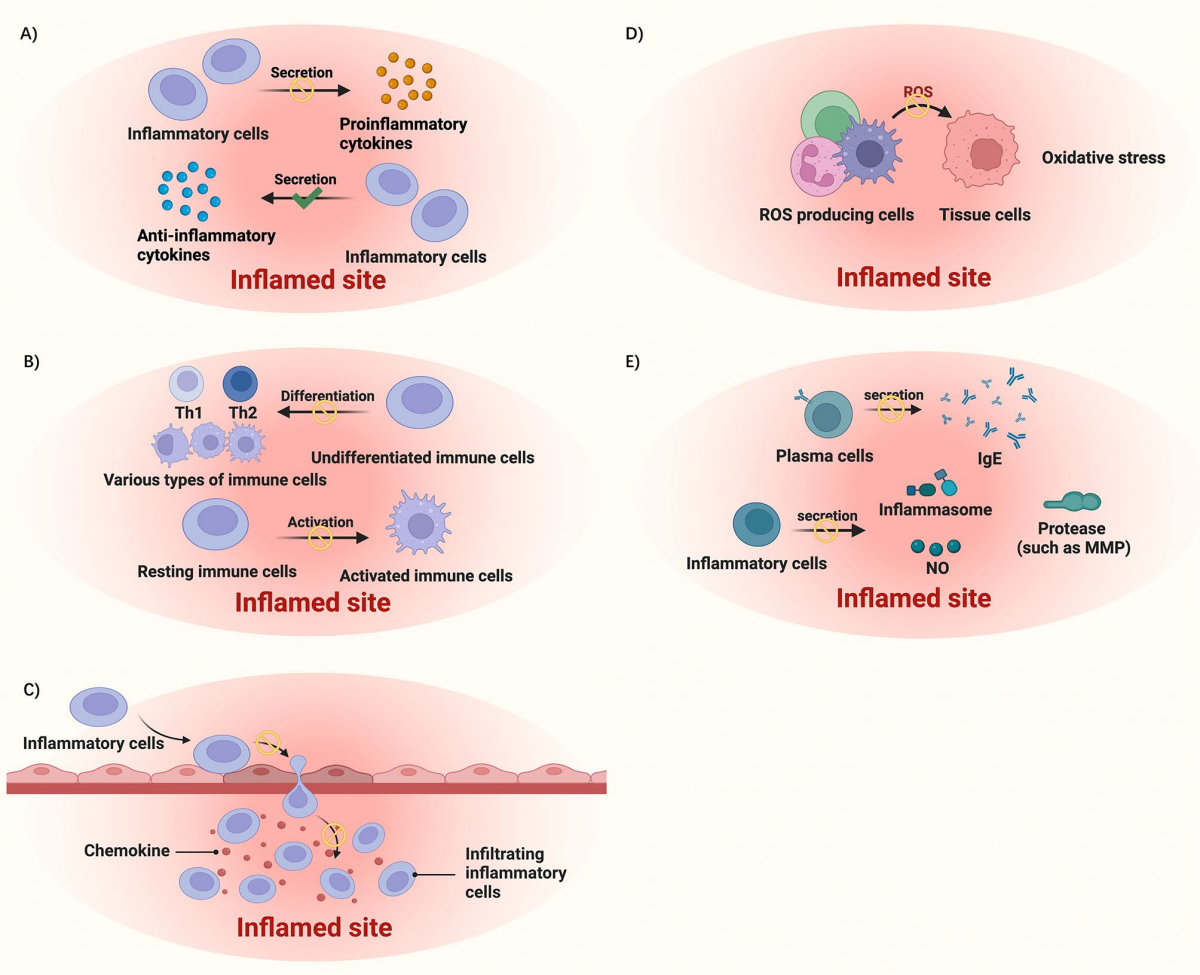
Anti-inflammatory mechanism of nanoparticles in the treatment of ocular diseases. (A) Nanoparticles promote the secretion of anti-inflammatory cytokines and inhibit the secretion of proinflammatory cytokines. (B) Nanoparticles inhibit abnormal activation and differentiation of inflammatory cells. (C) Nanoparticles inhibit the chemotaxis, recruitment and infiltration of inflammatory cells. (D) Nanoparticles inhibit abnormally activated oxidative stress. (E) Nanoparticles inhibit the secretion of other proinflammatory compounds.

### Improving drug corneal permeability

Drugs such as antibiotics and corticosteroids are commonly used for the anti-inflammatory treatment of infectious and noninfectious ocular diseases. According to the scope of action of these drugs, they can be divided into systemic treatments and local treatments. Topical treatments, such as eye drops, suspensions and ointments, are associated with fewer side effects than systemic treatments[5, 20, 21]. However, the corneal epithelium is the rate-limiting layer for hydrophilic drugs, and the corneal stroma is the rate-limiting layer for lipophilic drugs, resulting in limited corneal permeability. In most cases, less than 5% of these drugs reach the aqueous humor[5, 20, 22–26]. Regarding the problem of low corneal permeability, although nanoemulsions less than 1 μm in diameter have been shown to have the ability to penetrate the cornea, they cannot deliver water-sensitive molecules that are easily hydrolyzed[22]. At the same time, some drugs have limited water solubility, limiting their potential as eye drops; therefore, osmotic promoters, cell-penetrating peptides, and drug delivery systems emerged as potential treatments. However, osmotic promoters and cell-penetrating peptides can be cytotoxic at poorly controlled concentrations, so a more stable nanoparticle delivery system is preferable[27].

Nanoparticle delivery systems have great prospects in the treatment of ophthalmic diseases because of the small size, solubility and adjustable characteristics of NPs[7, 28]. Some NPs alter corneal permeability by disrupting the arrangement of phospholipids in the cornea. The monomer of 15-hydroxy stearate (Solutol® HS15) increases the corneal penetration of drugs, which may be associated with its distribution within the bilayer membranes of corneal epithelial cells. It can disrupt the order of phospholipid accumulation and create gaps that facilitate the passage of drugs. Adding coatings with a positive charge on the surface of NPs, such as chitosan (CHI) and L-arginine, is another effective strategy to improve permeability. For instance, Nirbhavane P et al. designed TA-loaded nanostructured lipid carriers (NLCs) with a positive cationic charge provided by stearic amine on the surface. They demonstrated that these TA-NLCs increased drug permeability by approximately 2 times and improved the intracellular uptake and retention of drugs[29].

### Reducing drug loss

At the same time, corneal areas and nasolacrimal duct drainage, blinking, tear flushing, and many other factors further reduce the penetration of drugs into the cornea. On the subject of drug loss, although ointments can improve drug bioavailability, the higher viscosity of ointments can also lead to greasy eyelids, enhanced blink reflexes, blur vision, and reduced patient compliance[5]. Similarly, in situ cementitious excipients and the incorporation of osmotic promoters can also promote drug absorption and function. However, the effect is slight and limited[24].

For certain drugs that degrade easily in vivo, slowing down the rate of degradation in vivo is a strategy. Polymers are options that can reduce unstable drug degradation and allow drugs to remain in extraocular tissues for extended periods. And they can interact more fully with corneal and conjunctival epithelial cells, resulting in increased bioavailability[22]. Carmelo Puglia et al. used NLCs with high drug packaging efficiency to load palmitoylethanolamide (PEA) to improve its stability and solubility in water, thereby increasing the ability of PEA to reach the retina. Furthermore, NLCs produced by a combination of high shear homogenization and ultrasonic (HSH/US) treatment showed excellent stability, which helped maintain the stability of the drug[30].

Increasing the viscosity of the administration system is a good strategy for reducing drug loss caused by tear flushing and drainage. The use of in situ gels (ISGs) is an excellent choice for this purpose. Owing to the properties of liquid dosage formulations with ISGs that can change into the gel phase when exposed to ocular physiological conditions, ISGs can be used to increase the retention time of drugs in the cornea and reduce drug loss[10, 12]. For example, Kexin Sun et al. found that the viscosity of a tacrolimus (TAC)-solid lipid NP (SLN) system did not increase with temperature changes. However, the viscosity of the TAC-SLN-ISG system increased obviously with increasing temperature, and eventually the system formed a rigid gel, indicating that the TAC-SLN-ISG system was a typical pseudoplastic system. In addition, the viscosity of a tranilast (Tra)-NP system was positively correlated with the concentration of ISG bases. The solubility of the Tra-NP system and its dispersivity in solution increased with the addition of an ISG base at 4 °C, which had a specific effect on reducing drug loss[12]. Similarly, the use of an SLN system has also been demonstrated to extend the corneal residence time of the drugs by improving the drug biocompatibility and mucosal adhesion properties[11].

### Prolonging the drug effective duration

Under current administration methods, such as eye drops, effective drug concentrations can remain sufficiently high at the site of the lesion for only a short time. Thus, administrations are frequently required to maintain effective drug concentrations at the target sites for long periods, which can reduce patient compliance and cause side effects such as ocular surface epithelial cell toxicity, corticosteroid-induced cataracts, and delayed epithelial healing[5, 20, 21]. For example, diclofenac sodium (DS), a nonsteroidal anti-inflammatory drug, is widely used to treat inflammation due to its potent inhibition of cyclooxygenase-2 (COX-2). However, the short half-life (approximately 2 h) of DS in vivo leads to the need for frequent administration to maintain a therapeutic concentration[31]. Similarly, most of the injected dexamethasone (Dex) is eliminated within 24 h, which means that frequent dosing is required to ensure an effective drug concentration[32].

Thus, ideas for the slow-release administration of drugs are urgently needed. Alternative ideas include loading drugs with biodegradable nanomaterials to achieve progressive drug release after material degradation, adding polyethylene glycol (PEG) coatings on the surfaces of NPs to delay drug release further, and adding special groups to NPs to control drug release patterns in specific environments.

In terms of biodegradable nanomaterials, the most commonly used nanomaterial is poly(lactide-co-glycolide acid) (PLGA). For example, Yung-Hsin Cheng et al. used biodegradable PLGA to load levofloxacin to prolong drug release and improve bioavailability[21]. Priyanka Bhatt et al. used PLGA to load resveratrol (RES) for the treatment of age-related macular degeneration (AMD), in which the release time of RES was maintained over 3 days[13]. A Alper Ozturk et al. used PLGA NPs loaded with DS for oral use to delay the rate of drug clearance. By plotting release curves, they demonstrated that DS was released from PLGA NPs in a bipolar pattern, characterized by sudden release in the first 3 h and continuous release in the following 24 h. In this system, drug release was driven by Fickian (pure diffusion) and non-Fickian release (due to relaxation of polymer chains between networks) mechanisms. In addition, they found that the release rate was directly proportional to the DS package size and inversely proportional to the size of the NPs[31]. Polycaprolactone (PCL)-Pluronic® F-68 (PF68) NPs also provide continuous drug delivery due to the slow biodegradability of PCL, with rapid release in the first 7 days and slow release lasting for 20 days [27]. The succinated triamcinolone acetonide (TA-SA)/poly(ethylene glycol)-poly(ε-caprolactone)-poly(ethylene glycol) (PECE) NPs designed by Jinhai Huang et al. also controlled drug release, showing sudden release in the first 8 h and continuous release in the following 72 h[33].

Dispersing the NPs in a hydrogel to deliver drugs would further slow the release rates of drugs. Sung-Hsin Cheng et al. developed a thermosensitive CHI-gelatin hydrogel containing curcumin (CUR) NPs and latanoprost to deliver both drugs simultaneously to extend the effective duration of a single administration. They observed that the release of CUR-NPs and latanoprost in hydrogels was prolonged for up to 7 days. The expression of tumor necrosis factor (TNF), interleukin (IL)-1α, IL-6, and matrix metalloproteinase (MMP)-13 in trabecular meshwork (TM) cells was subsequently downregulated by this administration method, and reactive oxygen species (ROS) production in mitochondria was significantly prevented[34]. In addition, Uri Soiberman et al. used NPs of a dendritic macromolecule composed of poly(amidoamine) (PAMAM) to load Dex and mixed the synthetic dexamethasone conjugate (D-Dex) into injectable gel preparations to achieve continuous release of D-DEX by utilizing the viscoelasticity of the gel. In in vitro experiments, the release of D-Dex from the gel lasted for approximately 96 h, and they suggested that the release of D-Dex could last for several weeks in a body with a low volume of fluid in contact with the gel[35].

Hydrophilic methoxy polyethylene glycol (mPEG) controls the biodegradation rates of PLGA. Therefore, the addition of PEG coating on the surfaces of biodegradable NPs is a potential method. Dadong Guo et al. used mPEG-PLGA NPs to load TA to achieve prolonged drug efficacy, which was finally proven to maintain drug release for 45 days[6]. Similarly, Sanchez-Lopez et al. added various surfactants, including a PEG coating based on PLGA. The use of PEG was beneficial to the sustained release process of drugs. Dexibuprofen (DXI) release was the fastest in 5% PEG NPs of PLGA, and the release slowed with increasing PEG concentration[23]. In addition, colloidal self-assembled patterns (cSAPs) formed by a combination of PLGA, L-histidine, and PEG have been shown to increase cell surface adhesion through charge conversion (moderately negative charge at pH 7.4 and converted to cationic charge at pH below 6.5 due to protonation of the poly(L-histidine) (PLH) imidazole group) and ring-opening reactions that release ultraviolet light (UV)-inactivated chlamydia trachomatis (Ct) (UV-Ct), thereby enhancing mucosal immunity in mice with long-term protective immunity[36].

Specific groups also show great potential for slowing down drug release. For instance, Dex-loaded NPs (PBA-GC@DEX NPs) designed by Yanlong Zhang et al. enabled the retention of the drugs in the retina for more than 2 days, showing a noticeable trend of slower drug release rates. They found that the cumulative release curve of DEX was related to the number of acrylamidophenylboronic acid (AAPBA) groups in sugar polymers. They suspect that increasing the number of AAPBA groups may increase the hydrophobic core of the NPs, which prevents drugs from leaking out of the NPs[32]. Liwen Wang et al. designed a new metal-organic framework (MOF), called His 6-metal assembly (HmA), to encapsulate dexamethasone sodium phosphate (Dexp), which showed excellent loading capacity, rapid endocytosis, intracellular release of loaded drugs, pH-dependent release, and low cytotoxicity. Most Dexp was released rapidly in acidic solution and was relatively stable in an alkaline environment, which might be related to the pKa of the diimidazole group of His 6 being 6.8. His 6 could not coordinate with Zn2 + due to its protonation in acidic solution. Hence, Dexp@HmA (Dexp in situ encapsulated into HmA) could be absorbed by various inflammatory cells in the cornea under alkaline conditions, with Dexp releasing slowly, which prolonged the effective duration of drugs [20].

### Establishing a targeting system

Conventional drug therapy often has the problems of nonspecific targeting, which results in drug waste and the need for larger doses. Excessive drug doses and off-target drug effects are more likely to cause adverse drug reactions. Therefore, a drug delivery system targeting the inflammatory site is worth developing.

The most straightforward method is to choose nanomaterials that can linger at the site of inflammation, including targeting inflammatory cells or inflammatory cytokines with nucleic acid fragments, short peptides, or charges on the surface of NPs. For example, PAMAM dendritic macromolecules are quickly cleared from targeted organs. They have the properties of selective localization and retention in inflammatory/injured areas, related to their localization to activated macrophages (Mps) at the site of injury[35, 37]. Rebecca G Edwards et al. used negatively charged 500 nm diameter immune modification particles (IMPs) derived from PLGA, which were absorbed by the macrophage receptor with collagen structure (MARCO) in an opsonin-independent manner. This process directed these inflammatory cells to the spleen and induced apoptosis, limiting tissue infiltration and protecting corneal health. In addition, they proposed that IMPs could inhibit the mobilization of immune cells to the cornea without blocking viral clearance, which made this an effective method for the treatment of chronic inflammation due to herpes simplex virus keratitis (HSK)[38]. Based on the characteristics of gold NPs (AuNPs), which are easily internalized by cells and tissues, Md Imam Uddin et al. constructed antisense sequence (AS)-vascular cell adhesion molecule (VCAM)-1 hAuNPs, which contained antisense sequences complementary to VCAM-1 mRNA, to aggregate to AMD-induced neovascularization. This system showed good binding to and enabled the imaging of VCAM-1 mRNA. VCAM-1 is an inflammatory marker that can be induced by the proinflammatory cytokine TNF-α. This binding site and binding mode provide an excellent reference for the targeted delivery of AMD-blocking drugs[39]. Kunbei Lai et al. then used ALA-Pro-ARg-Pro-Gly (APRPG), a short peptide that explicitly targets vascular endothelial growth factor receptor (VEGFR)-1, to address the low specificity of triptolide (TP). They used the nanoliposome-APRPG package TP for the treatment of choroidal neovascularization (CNV). They found that drug uptake by EA.hy96 endothelial cells increased after APRPG peptide modification[40].

Another idea is to search for some unique substances in the target site as targets for drug delivery. For instance, based on the fact that transferrin receptor (TfR) is widely expressed in the blood-eye barrier, R Ganugula et al. designed PLGA-gambogic acid (GA)2-CUR-NPs to improve the oral bioavailability of CUR using a noncompetitive active delivery strategy targeting TfR[41].

A better idea is to build a stimulus-triggered drug release system. Of all the forms of drug release by NPs, Fickian diffusion is the most common; however, it is not easy to regulate. In contrast, stimuli-responsive nanocarriers are an ideal drug release mode to mitigate the adverse effects of Fickian diffusion irregularity. Based on this target, Saad M Ahsan designed an inflammation-triggered gelatin nanostructure loaded with ketoconazole (KET), which was cleaved to release KET only under the action of proteases in the infectious microenvironment. The mechanism was that the anti-TLR4 antibody on the nanostructure surfaces bound to TLR4 on the human corneal epithelium (HCE), which was proportional to the degree of inflammation[42].

In addition to targeting sites of inflammation, drug delivery systems targeting the retina are also needed. It is difficult for conventional drugs to efficiently reach an inflamed retina because of its deep anatomical location. For example, intravitreal injection of corticosteroids (such as dexamethasone implants) can be used in some cases of diabetic macular edema (DME). However, the low permeability of these drugs makes it difficult to transport the drugs to the posterior eye. Although many experiments have been performed to test nanomaterials for drug transport, the membrane permeability of currently used nanomaterials is still relatively low. After subconjunctival injection, the effective doses of drugs reaching sites of retinopathy are still limited due to the barriers of the sclera and choroid[32, 43, 44]. Consequently, a carrier system with good water solubility and high stability to successfully target the retina is needed for drug delivery.

Amphiphilic NPs are good choices for these carrier systems. For example, Yanlong Zhang et al. used a nanoplatform composed of blocks of amphiphilic phenylboronic acid and atactic sugar polymers as a bioadhesion drug delivery system to efficiently transport Dex to the retina. They found that in addition to PBA-GC@DEX NPs having higher choroidal permeability than Dex, phenylboronic acid groups might bind to carbohydrates on the surface of a human retinal pigment epithelium (RPE) cell line (APRE-19) to facilitate cellular uptake of NPs. In addition, the presence of acrylamido glucopyranose (AGA) fragments could further improve the cellular compatibility of the drug delivery system[32]. Furthermore, it has previously been shown that NPs of approximately 200 nm in size with hydrophobic nuclei and mucosa-adhering hydrophilic shells can reach the retina. Based on this property, Binapani Mahaling et al. designed PLA core and CHI shell NPs to load azithromycin (AZM) and TA due to the hydrophilicity and mucosal adhesion of CHI. The results showed that the administration system played a good anti-inflammatory role in a mouse microglial cell line (BV-2) and significantly downregulated IL-1β[45].

### Delivering biologicals for the treatment of chronic recurrent and refractory ocular diseases

Vaccines represent a good strategy for the treatment of some refractory or relapsing ocular diseases. DNA vaccines have attracted extensive attention in infectious diseases due to their abilities to induce strong specific active antiviral immune responses. For example, ‘PRSC-Gd-IL-21’ (where Gd is an antigen and IL-21 is an adjuvant molecule) is a vaccine that was designed for the treatment of HSK. However, the vaccine itself still has shortcomings, such as weak immunogenicity[46]. At the same time, because of the uncertain safety, inadequate immunogenicity of candidate vaccines, lack of an effective vaccine delivery system, and lack of adjuvants for immune regulation, there is no acceptable vaccine for the treatment of some ocular diseases, such as human chlamydia [36].

Based on the properties of CHI NPs that have been shown to enhance the immune response induced by antigen, Li-li Dong et al. used CHI as the carrier of herpes simplex virus (HSV)-1 DNA vaccine and replaced the gD in the conventional DNA vaccine with glucocorticoids (GCs) combined with gD (Gd.GC) to enhance immunogenicity[47]. Similarly, Ru Tang et al. developed a PRSC-NLDC145.GD-IL21 DNA/CHI NP vaccine for recurrent HSK[46]. Both experiments successfully increased the robust humoral and cellular immune responses, including higher levels of specific neutralizing antibodies and sIgA, and enhanced spleen and NK-cell toxicity. Eventually, the incidence and symptoms in primary and relapsing HSK mice were both reduced significantly[46, 47]. Rajnish Sahu et al. then summarized the polymer NPs used in vaccines in recent years. PLGA NPs loaded with rat chlamydia major outer membrane protein (MOMP) could significantly increase the ratio of the Th1 antibody titer to the Th2 antibody titer, which reflected the self-adjuvant properties of PLGA polymer NPs. Poly lactic acid-co-poly ethylene glycol (PLA-PEG) is the formation of a PEG-surrounding poly lactic acid (PLA) core, which has the advantages of improving hydrophilicity, increasing drug loading capacity, delaying drug release, and delaying biodegradation. By encapsulating the recombinant peptides of MOMP (M278), PLA-PEG has been shown to activate the production of T-cell specific T helper (Th) 1 cytokines (interferon (IFN)-γ, IL-2), serum Th1 (IgG2a) and Th2 (IgG1, IgG2b) antibodies, which indicates an enhanced adaptive immune response[48].

In addition to vaccines, small interfering RNA (siRNA) delivery is also an advantage for NPs. Gene silencing induced by siRNA has shown great potential in treating chronic ocular diseases. The low dosage, blood‒retinal barrier obstruction, and inadequate lymphatic drainage keeps siRNA adverse reactions at low levels. However, it is still difficult to avoid potential adverse side effects caused by off-target effects and immunogenicity. Moreover, siRNA is not efficiently delivered to the posterior eye segment due to its high sensitivity to enzymatic hydrolysis, poor cellular absorption, and rapid elimination from the circulatory system. This means that siRNA delivery via a vector is the last resort. A virus is the most commonly used vector, but there are still some problems, such as high immunogenicity and high cost. Nonviral vectors, despite their low immunogenicity and cost, cannot reach all cell layers equally, making NPs the leading candidates for RNA carriers. Hence, Merve Sen et al. used magnetic NPs (MNPs) and magnetic forces to deliver valosin-containing protein (VCP) siRNA into retinal explants to target VCP, the therapeutic target of autosomal-dominant retinitis pigmentosa (adRP). They demonstrated that reverse magnetoreception of VCP siRNA was very effective[49].

### Establishing a non-invasive drug delivery system

Intravitreal injection of anti-VEGF drugs is a routine treatment for wet AMD since the formation and leakage of new blood vessels are the leading causes of injury. However, this is an invasive method of drug delivery that can lead to many complications, such as bleeding, pain, infections, and detachment. Therefore, a non-invasive drug delivery method needs to be developed.

NPs have considerably accelerated the achievement of the goal of noninvasive drug delivery due to their small particle sizes, enabling them to successfully penetrate the ocular surface barriers and reach the intraocular regions. Eye drops made of NPs are a promising drug for the treatment of inner ocular diseases[13, 14, 50].

## Mechanisms involved in the treatment of ocular diseases by nanoparticles

Inflammation is involved in the pathogenic process of many ocular diseases. Here, we divide the mechanisms into five categories according to the main proinflammatory components participate in and how they induce inflammation, including modulating cytokine production and secretion, modulating immune cell activation and differentiation, inhibiting immune cell infiltration, alleviating oxidative stress, and blocking the excretion of other inflammation-related molecules, as shown in Fig. 3.

### Modulating cytokine production and secretion

The regulation of cytokines is the most common immune mechanism of NPs for the treatment of ocular diseases. The mechanisms involved in the inflammatory pathogenic process include promoting the recruitment and activation of inflammatory cells and acting on immune cells to activate antigen presentation and the subsequent immune response. Based on the adverse effects of inflammatory factors, NPs inhibit the transcription of proinflammatory cytokines such as IL-1β, IL-6, IL-12, IL-17, IL-18, and TNF-α by blocking NK-κB expression, thereby reducing the secretion of these inflammatory cytokines[6, 12, 32–34, 45, 51–54]. In contrast, the expression and secretion of anti-inflammatory cytokines such as IL-10 and IFN-γ can be promoted by some NPs[10, 34, 51]. For example, Wang G et al. found that exosomes derived from adipose-derived stem cells (ADSC-Exos), which are natural NPs, can inhibit the production of IL-1β, IL-6, IL-1α, IFN-γ, and TNF-α and improve the expression of the anti-inflammatory cytokine IL-10[51].

### Modulating immune cell activation and differentiation

Another pathway by which NPs regulate inflammation is directly inhibiting the proinflammatory component-induced activation of immune cells and inducing the differentiation of immune cells from immune-killing to immune-protective subtypes. Here, immune cells can be divided into inflammatory cells (such as T cells) involved in specific and nonspecific immunity and other cells associated with inflammation (such as microglia)[22, 45, 49, 50, 55]. As Sun K et al. proved, TAC-SLNs successfully inhibited the process of the conversion of Th1 cells to Th2 cells and the activation of Th2 cells[10].

### Inhibiting immune cell infiltration

Abnormal infiltration of immune cells is detrimental to the recovery of inflammatory sites, which can be mediated by chemokines. Different types of chemokines showed certain determinative effects on the types of immune cells recruited, such as chemokine (C-X-C motif) ligand (CXCL) 1 and CXCL2 target chemokine receptor (CXCR) 2 on neutrophils, while CXCL10 targets CXCR3 on macrophages, dendritic cells (DCs) and activated T cells[56]. These infiltrating cells further secrete cytokines, proteases and free radicals to damage local tissues. NPs have been demonstrated to inhibit the aggregation of various immune cells at inflammatory sites, including macrophages, eosinophils, lymphocytes, and plasma cells [11, 14, 29, 33, 40, 57]. NPs may implement this feature by downregulating all kinds of chemotactic molecules[14, 39, 40, 58]. TP-Nanolip-APRPG, designed by Lai K et al., inhibited M2 Mp infiltration after downregulating ICAM-1 and monocyte chemotactic protein (MCP)-1 and finally reduced the infiltration rate in animal models[40].

### Alleviating oxidative stress

Both aging and sick conditions can generate ROS in the ocular regions. Excessive ROS can directly promote the death of RPE cells and cause damage to the blood‒retinal barrier by inducing an increase in VEGF. Inhibiting excessive oxidative stress, including blocking ROS formation and eliminating excessive ROS, is another important mechanism for the anti-inflammatory effect of NPs[50, 52, 53, 59]. Various NPs with reductive properties, such as selenium NPs (SeNPs), have been used to block mito-ROS production in RPE cells, thereby blocking oxidative stress-induced apoptosis of RPE cells[53].

### Blocking the excretion of other inflammation-related molecules

In addition to blocking the immune cells and inflammatory cytokines mentioned above, many other proinflammatory molecules secreted by inflammatory cells, including NO, inflammasomes, and proteases such as MMP-13, can also induce inflammatory damage by directly dissolving normal tissue, activating local intense inflammation. However, successful blocking may be facilitated by the rational use of NPs[12, 33, 34, 51, 57]. In addition, as important immune components, antibodies can be secreted by plasma cells. However, abnormal IgE secretion may result in a local hypersensitivity reaction, which has been successfully intercepted by TAC-SLNs[10]. Abnormal activation of the complement system (such as excessive production of complement C3) can also cause tissue damage, which can also be blocked by NPs[60].

## Ocular surface diseases

**Fig. 4:**
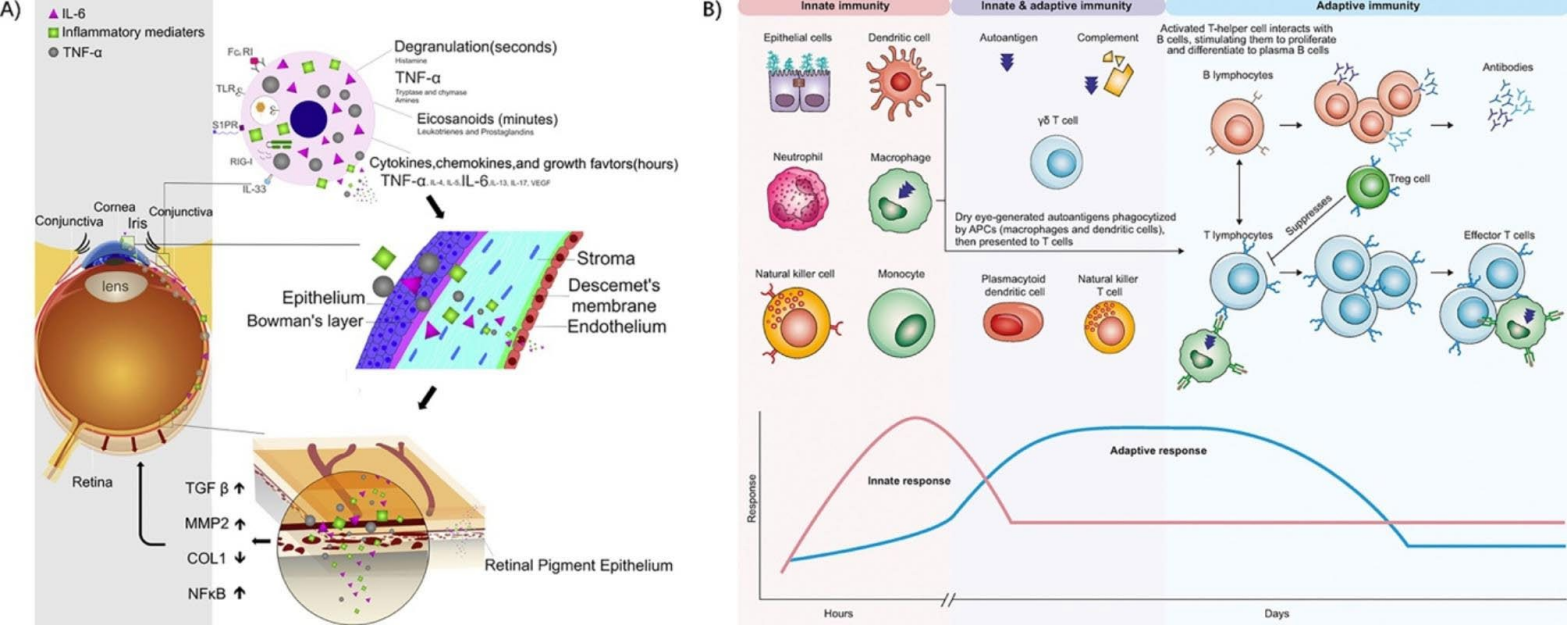
Model diagram of the inflammatory mechanism of ocular surface disease and the anti-inflammatory mechanism of nanoparticles. (A) Inflammatory signaling pathways involved in allergic conjunctivitis and ametropia induced by allergic conjunctivitis. (B) Major components of the immune response involved in acute DED. Copyright 2017, Royal Society of Chemistry. Reproduced under the terms of the CC-BY 4.0 license [73]. Copyright 2018, The Authors. Published by Elsevier. Reproduced under the terms of the CC-BY 4.0 license [77]. Copyright 2020, The Authors. Published by Elsevier.

The inflammatory mechanism plays an important role in various ocular surface diseases. Here, we summarize the inflammatory mechanism involved in the pathogenesis of various ocular surface diseases and the anti-inflammatory treatment mechanism of NPs in ocular surface diseases (as shown in Fig. 4; Table 1).

**Table 1:** The application of nanoparticles in ocular surface diseases

Citation	NPs	Diameter	Function of NPs	Anti-inflammatory mechanisms	Animal models	
[20]	Dexp@HmA	122 nm	Deliver Dexp	Enter inflammatory cells in the cornea to reduce the expression of IL-6 and TNF-α	Male Sprague‒Dawley rats with alkaline burn-induced keratitis	
[22]	TAC NPs	106–166 nm	Deliver TAC	Inhibit T-cell activation; reduce the expression of KC, MIP-2, IL-6 and GCSF; reduce the activity of polymorphonuclear cells and monocytes	Female C57BL/6 mice with LPS-induced keratitis	
[21]	Levofloxacin and PA-loaded PLGA NPs	154.7 ± 4.1 nm	Deliver levofloxacin and PA	Downregulate the expression of TNF-α, IL-6, MMP-3 and MMP-9	New Zealand albino rabbits with *Staphylococcus aureus*-induced keratitis	
[23]	DXI-PLGA NPs	200 nm	Deliver DXI	/	New Zealand albino rabbits	
[46]	DNA vaccine-CHI NPs	/	Deliver DNA vaccine	Induce a strong humoral immune response in mice with recurrent HSK, including high levels of sIgA and antibodies	BALB/c background female mice	
[11]	MZL-SLNs	172.53 ± 1.77 nm ~ 500.07 ± 10.6 nm	Deliver MZL	Reduce eosinophils and plasma cell infiltration, reduce VEGF and TNF-α levels	Albino rabbits with histamine-induced conjunctivitis	
[12]	Tra-NPs	40–190 nm	Deliver Tra	Inhibit the elevation of NO and TNF-α	Wistar rats with LPS-induced conjunctivitis	
[36]	PLA-PEG NPs, PLGA NPs, PLGA-PLH-PEG NPs	/	Deliver chlamydia antigen and antibiotics	Enhance cellular and humoral immunity against chlamydia, including increased production of some cytokines and antibodies	/	
[57]	Rapa-loaded FSI/FKBP-V48 NPs	FKBP-V48 3.6 nm, FSI 24 nm	Deliver Rapa	Reduce lymphocyte infiltration, downregulate most inflammatory genes, regulate the expression of mTOR-related genes, reduce the activity of CATS protease	NOD mice with LG dacryoadenitis	
[51]	mADSC-Exos	134 nm	Inhibit inflammation directly	Inhibit apoptosis; reduce the production of proinflammatory cytokines (IL-1β, IL-6, IL-1α, IFN-γ, and TNF-α); promote the production of the anti-inflammatory cytokine IL-10; reverse NLRP3 inflammasome activation; upregulate caspase-1, IL-1β, and IL-18	C57BL/6 mice with benzalkonium-chloride-induced DED	
[37]	D-Dex	~ 3–10 nm	Deliver Dex	Inhibit T lymphocyte infiltration, inhibit the expression of the inflammatory factors IL6, IL8, MMP9 and TNFα	New Zealand white rabbits with autoimmune lacrimal gland inflammation	

### Corneal diseases

Keratitis is one of the most common lesions of the cornea and is caused by weakened corneal defense and the invasion of endogenous and exogenous pathogenic factors into the corneal tissue. Keratitis can be divided into infectious and noninfectious keratitis according to pathogeny, and microbial keratitis can be further divided into bacterial, fungal, and viral infections[61]. Infectious keratitis caused by bacterial biofilms is one of the leading causes of corneal blindness[20, 62]. Fungal keratitis is mainly caused by trauma, a history of corticosteroid usage, contact lens wear and systemic or ocular defects. Fungal keratitis may be related to the upregulation of mannoglycoprotein in damaged corneas, which can be recognized and adhered to by mucins on the fungal surface[63, 64]. Herpes simplex keratitis is another cause of blinding keratopathy. This virus can travel in a retrograde manner to the trigeminal ganglion through the ophthalmic nerve, maintain a latent state, and then be reactivated under specific conditions[65]. Lingzhi Niu et al. noted that current treatments for fungal keratitis have limited efficacy[66].

#### Inflammatory mechanisms involved in the pathogenic process

The inflammatory mechanisms involved in infectious keratitis mainly include the production of numerous cytokines and chemokines, the involvement of nonspecific immune cells and specific immune cells, the existence of immune escape, etc. However, the specific mechanisms vary depending on the unique characteristics of different pathogens, such as specific components (e.g., bacterial lipopolysaccharide (LPS)) and specific host-invading processes (e.g., viral nucleic acid integration into the host nucleus).

LPS, a component of the cell wall of Gram-negative bacteria, can resist the killing effects of the host’s complement and activate the TLR 4 pathway to produce proinflammatory cytokines such as IL-6 and TNF-α, which recruit neutrophils for corneal infiltration, and lymphangiogenic factors, including VEGF-C and VEGFR-3, which are produced in large numbers in late keratitis. In lymphatic endothelial cells, TLR4 can further recruit macrophages. However, F4/80- and CD11b-positive macrophages can promote the formation of corneal lymphatic vessels, which is beneficial in reducing the severity of keratopathy in the late stage of bacterial keratitis[64, 67]. For HSK, inflammatory factors perform similar functions, and similar inflammation-related molecules are involved, including neutrophils producing cytokines, proteases, and free radicals to damage corneal tissue; various chemokines recruiting different types of immune cells; and TLR (represented by TLR-2, 4, 7, 8 and 9) regulating the immune response. Among them, TLR-4 showed beneficial effects against keratitis, while the rest promoted corneal injury[56, 68]. The capsule and hemolysin of *Streptococcus pneumoniae* are also common virulence factors. In addition to perforating host cells directly, hemolysin also activates complement system-induced inflammation. At the same time, *Acanthamoeba* trophozoites secrete a variety of proteases to nonspecifically decompose collagen tissue, including type I collagen (critical structural integrity of the cornea), sIgA, and other immunoglobulins (barriers for pathogenic microorganisms)[64].

Immunosuppression and immune escape also play an important role in the pathogenesis of keratitis. Bacterial exopolysaccharides, another component of bacteria, can inhibit local immune responses[64, 67]. The MucD protease of *P. aeruginosa* has been shown to inhibit corneal neutrophil recruitment and secretion of IL-1β and macrophage inflammatory protein (MIP)-2, which helps *P. aeruginosa* achieve immune escape[64]. In addition, HSV-1 enters the body by various means of immune escape, including the formation of infected corneal epithelial cell syncytia to avoid immune attack during cell-to-cell spread, ubiquitination of specific host proteins (such as interferon-inducible protein (IFI)), phosphorylation of targeted transcription factors to interfere with nuclear translocation and the expression of target genes, and delivery of major histocompatibility complex (MHC)-I antigens. In general, after HSV infection, a normal immune system can still effectively limit the transmission of the virus, but when immune function is suppressed, viral replication will be enhanced, and the virus will eventually invade the stroma, causing HSK[56].

Autophagy, apoptosis and inflammation are often closely related and mutually regulated. MiRNAs have been proven to regulate autophagy by influencing inflammation, cell proliferation, and apoptosis. Therefore, autophagy disorder is not only the intermediate link of the pathological changes caused by various ocular diseases but also the cause of a variety of ocular diseases through inflammatory mechanisms. In fungal keratitis, miR-665-3p overexpression can not only increase the production of IL-1β but also inhibit autophagosome formation (i.e., impaired autophagic flux) by targeting autophagy-related (ATG) 9 A, ATG14, ATG4B and ATG5 genes, among which ATG5 is most closely related to autophagosome formation[69]. However, in mice with ATG5-deficient viral keratitis, it has been demonstrated that blocking DC autophagy reduces the severity of the disease[56].

The upregulation of VEGF promoted by IL-6 and Th17-secreted IL-17, combination of IFN-γ from Th1 cells and IL-2 to enhance antigen presentation, and induction of epithelial cell apoptosis by IFN-γ and granase secreted after natural killer (NK) cell activation are all involved in the mechanisms of keratitis[56, 68]. In addition to the direct dissolution and phagocytosis of host cells, the pathogenesis of *Acanthamoeba* infection also includes the production of MIP133 after mannose stimulation and the activation of corneal epithelial cell apoptosis by a caspase-3-dependent pathway[64].

#### Anti-inflammatory effects of nanoparticles

In addition to inhibiting cytokine production, some nanoplatforms also inhibit the recruitment and infiltration of inflammatory cells at the inflammatory site.

Various types of NPs, including PAMAM dendritic molecules, MOFs, polymers, etc., have been used to deliver drugs to treat keratitis. They are combined or added with certain surface coatings to achieve better drug delivery and more significant downregulation of cytokines and chemokines. For example, Yung-Hsin Cheng et al. designed a dual-drug delivery system consisting of prednisolone-loaded chitosan hydrogel and levofloxacin-loaded PLGA. This administration can effectively downregulate the expression of inflammatory genes related to TNF-α, IL-6, MMP-3 and MMP-9, which benefit from the sustained-release effect of this administration system[21]. Saad M. Ahsan designed a gelatin nanostructure loaded with KET, which was cleaved to release KET only under the action of proteases in an infectious microenvironment. The results showed that the nanostructure could significantly reduce the mRNA levels of IL-8, TNF-α, and MMP-2[42]. In addition, based on the nanoscale, polyvalence, monodispersity, high water solubility, multiple hydroxyl groups on the surface of the accessible introduced drugs, and properties of PAMAM, Uri Soiberman et al. found that dendritic macromolecules specifically targeted activated Mps in the center of corneas burned by alkali. Finally, D-DEX significantly reduced MCP-1 mRNA and VEGF mRNA levels and promoted the regression of corneal edema after alkali burns[35]. Sanchez-Lopez et al. added a PEG coating to DXI-PLGA to promote the corneal infiltration of NPs, which has been demonstrated to further reduce inflammation scores. Other surfactants, such as Tween, could also increase the anti-inflammatory effect due to their ability to increase membrane permeability[23]. Based on the advantages and disadvantages of MOFs, Liwen Wang et al. designed a new MOF called HmA to encapsulate Dexp, which had excellent loading capacity, rapid endocytosis and intracellular release of loaded drugs, pH-dependent release, and low cytotoxicity. After ocular medication, Dexp@HmA was first absorbed by various inflammatory cells in the cornea under alkaline conditions, with Dexp releasing slowly, which prolonged the time in which an adequate concentration of drug was present. Subsequently, with endosomes formed after endocytosis undergoing acidification during lysosomal maturation, Dexp was released in large quantities to rapidly and effectively reduce inflammation. The results showed that IL-6 and TNF-α levels were significantly reduced[20].

In inhibiting the local infiltration of inflammatory cells, MIP-2 was significantly elevated in LPS-induced keratitis, which could recruit polymorphonuclear cells toward the corneal stroma as a functional analog of IL-8. These recruited immune cells produced ROS and MMPs to destroy the corneal stroma and reduce corneal clarity. Therefore, Rebibo L et al. encapsulated TAC, a macrolide that inhibits calcineurin activity in T cells, into nanocapsules to reduce the production of cytokines and inflammatory symptoms in patients with keratitis. The results showed that T-cell activation, the expression of MIP-2, IL-6 and granulocyte colony-stimulating factor (GCSF), and the activity of polymorphonuclear cells and monocytes were inhibited by the nanocapsules[22]. Herpes virus entry mediator (HVEM), a cell receptor, has been shown to facilitate the entry of HSV-1 into cultured human corneal epithelial cells (HCEs), human conjunctival epithelial cells, primary human monocytes, and DCs and is also closely involved in the production and recruitment of inflammatory cytokines and inflammatory cells. The increased expression has been shown to lead to a loss of corneal sensitivity and leukocyte infiltration. Hence, HVEM is one of the targets for preventing and treating HSV-1-induced viral keratitis. In addition to gene ablation, Rebecca G Edwards et al. used negatively charged 500 nm diameter IMPs derived from PLGA. They first found that most HVEM + cells in the cornea during acute infection were derived from the monocyte/macrophage lineage, in which negatively charged IMPs were absorbed by the MARCO receptor in an opsonin-independent manner. This process directed these inflammatory cells to the spleen and induced apoptosis, limiting tissue infiltration and protecting corneal health. In addition, IMPs could inhibit the mobilization of immune cells to the cornea without blocking viral clearance, making it an effective method for treating HSK chronic inflammation[38].

Contact lenses, a significant cause of corneal infections, can also be modified to reduce the risk of keratitis. Xiaoqi Liu et al. deposited an AgNP-containing polydopamine (PDA) coating on the surface of contact lenses to carry and release drugs at a sustained rate to treat keratitis with few side effects. To avoid an insufficient amount of AgNPs loaded on the coating and to reduce the light transmittance of the PDA coating after secondary oxidation by the conventional impregnation method, they prepared AgNPs by reducing Ag + with excessive dopamine. In this case, the excess dopamine could self-polymerize on AgNPs and contact lenses. Eventually, strong connections were formed between AgNPs and contact lenses. They found that the AgNPs were cytotoxic to adherent Mps, which might be associated with Ag-S bond formation in cells and AgNP-generated ROS toxicity to Mps[70].

Vaccines represent another alternative treatment or prevention strategy for HSK, which is challenging to treat and prone to recurrence. DCs are responsible for initiating specific immune responses during corneal infections. The unique and highly expressed DEC205 in DCs is one of the endocytosis uptake receptors, and it can be used as a target molecule for vaccines. Based on the properties of CHI NPs, which have been shown to enhance the immune response induced by antigens, Ru Tang et al. developed a PRSC-NLDC145.GD-IL21 DNA/CHI NP vaccine for recurrent HSK[46]. Similarly, Li-li Dong et al. used CHI as the carrier of the HSV-1 DNA vaccine and replaced the gD in a conventional DNA vaccine with Gd.GC to enhance immunogenicity[47]. Both experiments demonstrated that CHI NPs could induce more robust humoral and cellular immune responses, including higher levels of specific neutralizing antibodies and sIgA, and enhanced spleen and NK-cell toxicity. Eventually, the incidence of HSK in mice was successfully reduced and symptoms were relieved in primary and relapsing HSK mice[46, 47].

### Conjunctivitis

Conjunctivitis can be caused by a weakened ocular surface defense or enhanced external pathogenic factors, with conjunctival vascular dilation, exudation, and cell infiltration as the main pathological manifestations. Similarly, conjunctivitis is divided into infectious conjunctivitis and noninfectious conjunctivitis. According to the pathogen, infectious conjunctivitis can also be divided into bacterial conjunctivitis, viral conjunctivitis, and fungal conjunctivitis. The immune mechanism of allergic conjunctivitis is characterized by IgE-mediated mast cell threshing and T lymphocyte-mediated immune hypersensitivity. Although conjunctivitis rarely leads to permanent vision loss or structural damage, its cost is still high in terms of diagnosis and treatment, time, and money[71, 72].

#### Inflammatory mechanisms involved in the pathogenic process

Acute conjunctivitis (AC), a type 1 hypersensitivity reaction, is caused by Ig-E-mediated mast cell threshing and a T-cell-mediated immune response. When the sensitizer is processed into a sensitizing peptide, it can be delivered with the assistance of MHC-II. During this process, the levels of a large number of inflammatory cytokines are regulated (including the upregulation of TNF-α, IL-4, IL-6, IL-8, IL-13, MCP-1 and NF-κB and downregulation of IL-10 and κB inhibitors), and a large number of nonspecific immune cells infiltrate (including neutrophils, lymphocytes, macrophages, and eosinophils)[60, 72, 73]. Among them, IL-4 can promote IgE production by B cells to bind to basophils and mast cells, which will quickly target allergens and be activated upon re-exposure[72].

When the disease progresses to chronic stages, such as vernal keratoconjunctivitis (VKC), mast cells and eosinophils can degranulate and release cytokines, resulting in severe corneal ulceration, pruritus, and even the formation of large papillae at the corneal limbus[72]. The eosinophils mentioned here then promote the recruitment and activation of neutrophils[60]. Interestingly, it has been found that higher-than-normal innate immunity, represented by the overproduction of neutrophils, could block the meibomian gland and lead to meibomian gland inflammation[74]. Both ICAM-1 and human leukocyte antigen (HLA)-DR were found in conjunctival epithelial cells during the chronic pathological process. Inflammatory factors show different secretion statuses for different types of chronic AC. For example, granulocyte-macrophage colony-stimulating factor (GM-CSF) is elevated in all chronic AC types, IL-3 is expressed only in atopic keratoconjunctivitis (AKC) and VKC, Th1 cytokines are mainly expressed by AKC patients, and Th2 cytokines are mainly expressed by VKC patients[75]. The activation of the complement system is also involved in the pathogenesis of giant papillary conjunctivitis. Decay accelerating factor (DAF), which inhibits C3 complement activation, was inhibited. Moreover, leukotriene C4 expression was increased. In terms of specific immunity, M cells present in conjunctiva-associated lymphoid tissue (CALT) of upper meibomian tissue take up antigens and transport them to upstream immune cells (such as B cells), which activates the immune response[60].

#### Anti-inflammatory effects of nanoparticles

The treatment of immune conjunctivitis is a research hotspot in the field of NPs. The mechanisms involved mainly inhibit the overexpression of inflammatory factors, the production of other proinflammatory substances (such as NO), the oversecretion of pathological antibodies and the differentiation of inflammatory cells.

Kexin Sun et al. demonstrated that TAC-SLNs improved the efficacy of TAC, including downregulating serum IL-4, inhibiting the synthesis of IgE, inhibiting the activation and degranulation of conjunctival mast cells and the release of inflammatory mediators, and inhibiting the transformation of Th1 to Th2 cells by upregulating IFN-γ, which is beneficial for the control of type I hypersensitivity of immune conjunctivitis[10]. Similarly, El-Emam GA et al. found that the presence of SLN promotes mizolastine (MZL) to inhibit eosinophil and plasma cell infiltration and reduce the levels of VEGF and TNF-α[11]. Misa Minami et al. selected Tra, an antiallergic drug widely and safely used in ophthalmology due to its ability to inhibit the synthesis of transforming growth factor (TGF)-β1 in various cells, thereby inhibiting the accumulation of collagen in granulation tissues and the release of chemical mediators in mast cells, as the therapeutic drug for conjunctivitis. They loaded Tra within NPs and modified them by various ISGs to form Tra-NP-incorporated ISNGs. As a result, Evans blue (EB) exudation, NO levels, and TNF-α levels decreased after treatment with Tra-NP-incorporated ISNGs, especially at 5 h after nTra-F68/F127-L infusion, which showed a significant preventive effect against conjunctivitis. Nevertheless, low ISG base and high ISG base Tra preparations showed no significant difference in the inhibition of NO and TNF-α[12].

Polymeric NPs are potentially effective vaccine carriers and adjuvants due to their biocompatibility and biodegradability. Rajnish Sahu et al. then summarized polymeric NPs used in vaccines in recent years. As mentioned above, PLGA NPs loaded with MOMP enhance adaptive responses with high biocompatibility and bioavailability, including the activation of Th1 cytokines and Th1 and Th2 antibodies[48]. In addition, cSAPs formed by a combination of PLGA, L-histidine and PEG have been shown to increase cell surface adhesion through charge conversion (moderately negative charge at pH 7.4 and converted to cationic charge at pH below 6.5 due to protonation of the PLH imidazole group) and ring-opening reactions that release UV-Ct, thereby promoting IFN-γ production, a robust antibody response, and CD4 + T-cell activation, which demonstrated enhanced mucosal immunity in mice with long-term protective immunity[36].

### Dry eye diseases

Sjogren’s syndrome (SjS) is a chronic autoimmune disease characterized by lymphocytic infiltration and a loss of function of the lacrimal gland (LG) and salivary gland (SG), which can be clinically characterized as dry eye disease (DED). DED is a multifactorial disease characterized by a persistently unstable and deficient tear film (TF) causing discomfort and visual impairment, accompanied by variable degrees of ocular surface epitheliopathy, inflammation and neurosensory abnormalities, according to a clinical consensus presented after four expert meetings[76].

#### Inflammatory mechanisms involved in the pathogenic process

The process of DED caused by SjS involves multiple inflammatory mechanisms, including epithelial cells and other innate cells (neutrophils, monocytes, Mps, and DCs) triggering flares as osmolarity increases. Over time, the downstream signaling pathway is activated and produces an adaptive immune response, which causes inflammation[77]. Pathogenic T cells usually infiltrate the ocular surface tissues of patients with chronic DED. Furthermore, a large number of proinflammatory factors, such as TNF-α, IL-1 α, IL-1 β, IL-6, IL-13, INF-γ and MIP-1α, can be found in the ocular surface tissue and tears of patients with DED[51]. The increase in IFN-γ plays a significant role in injuring the lacrimal gland by activating many inflammatory pathways. IFN-γ is mainly derived from activated NK cells, T cells and B cells. It can induce the differentiation of naive CD4 + T cells into the Th1 type, stimulate the expression of MHC II, and reduce some components in tears (including Rab3 D). In addition, the IFN signaling pathway, IRF 5 signaling pathway, lymphocyte signaling pathway, lymphocyte function-associated antigen (LFA)/ICAM-1 signaling pathway and NF-κB signaling pathway all play roles in SjS[78]. Furthermore, NOD-, LRR- and pyrin domain-containing 3 (NLRP3) inflammasome imbalance, which may be closely associated with a hypertonic environment, can lead to the release of IL-1β and IL-18 and subsequently increased inflammation, so the inhibition of NLRP3 can alleviate dry eye symptoms and ocular surface inflammation[51].

#### Anti-inflammatory effects of nanoparticles

In addition to commonly blocking the production of inflammatory factors and inflammasomes and inhibiting lymphocyte infiltration, the inflammatory strategies used in the treatment of DED also include specifically targeting some inflammatory cells and regulating the expression of inflammation-related genes.

As early as 2013, Mihir Shah et al. designed elastin-like polypeptide (ELP) NPs consisting of (Val-pro-Gly-Xaa-Gly) N pentamer repeats to load rapamycin (Rapa) to achieve slow release and reduce renal exposure to drugs, including FSI (48 ELP repeats with Xaa = Ser and 48 ELP repeats with Xaa = Ile) and FKBP-V48 (48 ELP repeats with Xaa = Val). The histological results demonstrated that FSI-Rapa reduced the area of lymphocyte infiltration by approximately 50%. In addition, they found that 40 of 84 essential genes associated with cytokines and chemokines were altered in FSI-Rapa-treated mice, while only 18 genes were affected in Rapa-free mice. The genes involved overlap but were not identical. The mechanistic target of the rapamycin (mTOR) gene could be upregulated by free Rapa, which may reduce efficacy, while the FSI preparation did not cause mTOR upregulation[57]. Hui Lin et al. used PAMAM dendritic macromolecules with a clear structure, small size, excellent water solubility and high safety for Dex transportation. The properties of PAMAM dendritic macromolecules were easily removed from target organs, localized and retained at sites of inflammation or injury enable them to activate Mps where injury occurs. Hui Lin et al. conducted an experiment with New Zealand white rabbits with autoimmune lacrimal gland inflammation. The results showed that D-DEX successfully inhibited T lymphocyte infiltration and the expression of the inflammatory factors IL6, IL8, MMP9 and TNFα[37]. Recently, Su Li et al. from Zhejiang University developed a Tempo (Tem)-conjugated cationic polypeptide micelle eye drop to treat DED. Excellent ROS scavenging ability in vitro, inhibition of NF-κB p-p65, MMP-9, IL-1β, and GAPDH protein expression, and reversal of M1 macrophage overactivation contribute to its function of improving tear film stability and promoting tear production[79].

Guifang Wang et al. investigated the efficacy of ADSC-exos, a type of natural nanoparticles, in treating DED based on the anti-inflammatory and immunomodulatory properties of adipose-derived mesenchymal stem cells (ADSCs) through the paracrine action of bioactive factors. It was shown that ADSC-exos successfully reduced the production of the proinflammatory cytokines IL-1β, IL-6, IL-1α, IFN-γ, and TNF-α, promoted the production of the anti-inflammatory cytokine IL-10, and reversed NLRP3 inflammasome activation and upregulation of caspase-1, IL-1β, and IL-18. Finally, the stability of the tear film was increased, and corneal surface injury was reduced[51].

## Vitreoretinal diseases

**Fig. 5:**
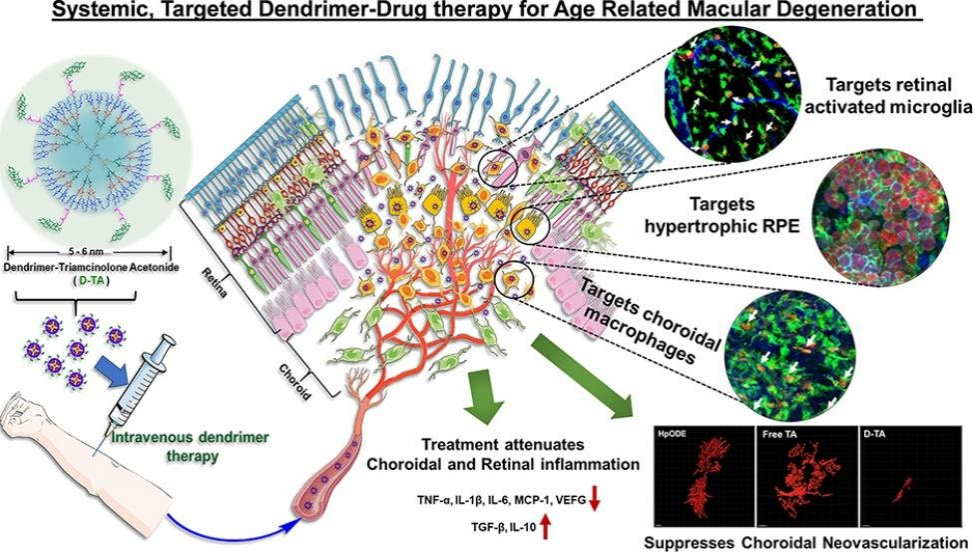
Model diagram of the inflammatory mechanism of ocular vitreoretinal disease and the anti-inflammatory mechanism of nanoparticles. Therapeutic targets of intravenous dendrimer therapy and the regulation of inflammatory factors in AMD patients. D-TA targets activated microglia, RPE cells and macrophages in the retina. The proinflammatory cytokines TNF-α, IL-1β, IL-6, MCP-1 and CEGF are downregulated, and the anti-inflammatory factors TGF-β and IL-10 are upregulated. Reproduced with permission [14]. Copyright 2021, Elsevier.

Here, we summarize the inflammatory mechanism involved in the pathogenesis of various vitreoretinal diseases and the anti-inflammatory mechanism of NPs in the treatment of vitreoretinal diseases (as shown in Fig. 5; Table 2).

**Table 2:** The application of nanoparticles in vitreoretinal diseases

Citation	NPs	Diameter	Function of NPs	Anti-inflammatory mechanisms	Animal models	
[30]	PEA-NLC	/	Deliver PEA	Downregulate TNF-α in retinal tissue	Sprague‒Dawley rats with STZ-induced diabetes	
[58]	RES-AuNPs	10 mm	Deliver RES	Reduce pigment epithelium-derived factor and VEGF-1; reduce the expression of TNFα, MCP-1, ICAM-1, IL-6, and IL-1β; reduce the phosphorylation of NF-κB P65; block the ERK1/2 signaling pathway	Wistar rats with STZ-induced DR	
[32]	PBA-GC@DEX NPs	120–180 nm	Deliver DEX	Downregulate the expression of IL-6 and TNF-α	Rats with STZ-induced DR	
[53]	SeNP	30 ~ 60 nm	Inhibit inflammation directly	Inhibit the increased fluorescence intensity of [Ca 2+] C in ARPE-19 cells induced by TRPM2 activation; decrease TRPM2 current density; decrease mito-Depol, cell solute and mito-ROS production in ARPE-19 cells; downregulate TNF-α and IL-1β levels; downregulate PARP-1 and TRPM2 in ARPE-19 cells	/	
[49]	siRNA/MNP	180 nm	Deliver siRNA	Silence VCP in retinal cell lines, reduce microglial activation	Homozygous P23H rhodopsin transgenic rats	
[59]	OCCNPs	100 nm	Inhibit inflammation directly	Inhibit the oxidative stress response; inhibit the apoptosis of ARPE-19 cells; downregulate the expression of the inflammatory factors TNF-α and IL-6	/	
[14]	D-TA	5.4 ± 0.2 nm	Deliver TA	Decrease the expression of proinflammatory factors (TNF-α, IL-6, IL-1β, MCP-1), inhibit the secretion of VEGF-165, inhibit macrophage infiltration	SD rats with 13(S) HpODE-induced CNV	
[13]	RES-loaded PLGA NPs	102.7 ± 2.8 nm	Deliver RES	Downregulate NF-κB and HIF-1α transcription factors to inhibit the expression of VEGF	/	
[39]	AS-VCAM-1 hAuNP	15 nm ~ 20 nm	Inhibit inflammation directly	Target to the inflammatory marker VCAM-1	C57BL/6J male mice with laser-induced CNV	
[40]	TP-nanolip-APRPG	123 nm	Deliver TP	Decrease the expression of VEGF, downregulate ICAM-1 and MCP-1, inhibit M2 macrophage infiltration	C57BL/6J mice with CNV induced by laser	
[45]	PLA-AZM NPs, PLA-TCA NPs	200–250 nm	Deliver AZM and TCA	Target BV-2 microglia, inhibit the expression of TNF-α and IL-1β	C57BL/6J mice	
[54]	AuAgCu2O-BS NPs	190 nm	Deliver BS	Downregulate elevated IL-1β and IL-6	New Zealand white rabbits with MRSA-induced endophthalmitis	

### Diabetic retinopathy

Diabetic retinopathy (DR) is the most common retinal vascular disease caused by inflammation, retinal ischemia, hypoxia (HYPX), the deposition of advanced glycation end-products (AGEs), the overexpression of VEGF, and elevated levels of interleukin or ROS[80]. According to pathological changes, DR can be divided into proliferative retinopathy and nonproliferative retinopathy. DR can be further divided into mild DR, moderate DR, and severe DR according to the severity of the disease.

#### Inflammatory mechanisms involved in the pathogenic process

In the DR model, many proinflammatory cytokines (IL-1β, TNFα, IL-8, IL-6, and MCP-1) and proteins (endothelin 1, sE-selectin, VEGF, ICAM-1, and CXCL10/IP) can be upregulated. Among them, IL-1β activates NF-κB through the JNK and P38 MAPK pathways, thereby inducing the secretion of other proinflammatory factors[81].

Oxidative stress is also involved in the pathogenesis of DR and can crosstalk with inflammation. In addition to four primary metabolic disorders (the activation of the protein kinase C pathway, polyol pathway flux, hexosamine pathway and intracellular formation of advanced glycation end-products), abnormal activation of NF-κB, decreased Nrf2 activity, and mitochondrial dysfunction induced by hyperglycemia can also induce ROS overproduction. NF-κB promotes inflammation by driving NO production and promotes apoptosis by activating MMP-9. Diabetes itself, of course, upregulates iNOS, a proinflammatory protein. High blood glucose levels can also lead to abnormal modifications of histones or transcription factors (such as changes in methylation levels) and abnormal expression of noncoding RNAs (including miRNAs and lncRNAs), which can also lead to the overexpression of proinflammatory proteins and the inhibition of ROS clearance. With the upregulation of ROS, VEGF is increased and then damages the blood‒retinal barrier. At the same time, prostaglandin E_2_ (PGE_2_), COX-2 and the activation of leukocytes can also increase VEGF and aggravate inflammation[81–83].

Capillary formation can increase the density of acellular capillaries and the number of TUNEL-positive capillaries in the retina[81, 84]. TNF-α can promote the adhesion of leukocytes in the blood vessels and the permeability of leukocytes by affecting the expression of ZO-1 and occludin. IL-6 can lead to vascular endothelial injury by upregulating ICAM-1 and downregulating ZO-1 and occludin. IL-8 recruits neutrophils and monocytes to increase the level of inflammatory cells in vitreous fluid, thereby promoting the formation of new blood vessels. In addition, all these proinflammatory cytokines can activate microglia, promoting VEGF secretion[81].

High blood sugar levels have also been shown to stimulate the activation of leukocytes and lead to leukocyte adhesion and leukocyte leakage by increasing ICAM-1 and integrin alpha 4/CD49d on vascular endothelial cells[81–83]. There are fewer Treg cells and more senescent T cells, indicating reduced immune homeostasis, which can still deposit and be activated by DCs[60a]. Furthermore, the complement systems can be widely activated, which is manifested by the deposition of the final product of complement activation (C5B-9) and the increased content of complement C3 and complement factor I[82].

#### Anti-inflammatory effects of nanoparticles

The inflammatory mechanisms involved in the treatment of DR by NPs include reducing excessive oxidative stress, reducing the excessive formation of cytokines and blocking the activation of inflammatory signaling pathways.

A variety of NPs have been used as drug carriers to aggregate drugs to effective concentrations in the retina. For example, Carmelo Puglia et al. used NLCs with high drug packaging efficiency to load PEA to improve its stability and solubility in water, thereby increasing the ability of PEA to reach the retina. The results showed that PEA-NLCs achieved detectable levels in retinal tissue and successfully downregulated the expression of TNF-α[30]. Similarly, Binapani Mahaling et al. used PF68 as a shell for PCL NPs based on the high solubility and strong permeability of PF68 to improve the TA concentrations in the retina. As expected, the NPs successfully reduced NF-κB expression and nuclear translocation, as well as TNF-α levels[27]. Yanlong Zhang et al. used a nanoplatform composed of blocks of amphiphilic phenylboronic acid and atactic sugar polymers as a bioadhesion drug delivery system to efficiently transport Dex to the retina. They found that in addition to PBA-GC@DEX NPs having higher choroidal permeability than Dex, phenylboronic acid groups might bind to carbohydrates on APRE-19 cell surfaces to facilitate cellular uptake of NPs. In addition, the presence of AGA fragments could further improve the cellular compatibility of the drug delivery system. The results showed that this approach could significantly downregulate the levels of IL-6 and TNF-α in the retina[32].

In addition to drug delivery, some NPs can also have anti-inflammatory properties, including the downregulation of inflammatory cytokines and chemokines and the blockade of inflammatory signaling pathways. Trans-3,5,4’-trihydroxystilbene is thought to play a therapeutic role due to its vascular protective and antioxidant properties. As a result, RES was used to synthesize AuNPs without a toxic reducing agent. The AuNPs significantly decreased IL-6 mRNA and IL-1β mRNA expression in the retina and VCAM-1, ICAM-1, and MCP-1. Furthermore, the NF-κB and ERK 1/2 signaling pathways were also blocked[58]. HYPX can induce the excessive formation of ROS, leading to oxidative damage and cell death, which is involved in the pathophysiological process of many ocular diseases, including DR. During this process, ARPE-19 cells expressed more inflammatory mediators, such as TNF-α and IL-1β. Antioxidant trace elements have been used to treat such oxidative damage, but there is a problem of proximity between toxic dose and therapeutic dose. Antioxidant NPs control the toxicity of trace elements. Dilek Ozkaya et al. then used SeNPs for research. They demonstrated that SeNPs block HYPX-induced transient receptor potential melastatin 2 (TRPM2) activation, ARPE-19 cell death, ROS production, and inflammatory cytokine elevation[53].

### Retinitis pigmentosa

Retinitis pigmentosa (RP) is a collective name for a group of genetic diseases that cause rod cells to die and cause secondary cone death. AMD and RP are considered the two most common degenerative diseases of the retina[84]. The genetic mechanisms of some RPs involve mutations in pre-mRNA processing factors such as SNRNP200, PRPF3/4/6/8/31 and RP9. The progressive degeneration of photoreceptors and the RPE is the characteristic change of RP. With the progression of the disease, night blindness caused by rod cell damage, a loss of central vision caused by cone degeneration, and eventually blindness will appear in sequence[85].

#### Inflammatory mechanisms involved in the pathogenic process

Microglia are activated in the presence of inflammation and injury. RIP1/RIP3/DRP1 axis-mediated necrotizing apoptosis leads to rod cell death, which induces the activation of IL-1β-secreting microglia and the upregulation of the Nlrp3 inflammasome, caspase 1 (CASP1), IL-1β, and IL-1RA. These inflammatory mechanisms can cause photoreceptor cell death and RP[84]. Although the activation of microglia itself does not induce the degeneration of cones, it was found that the inhibition of microglial activation had protective effects on cones. In addition, it has been demonstrated that a high-fat diet can induce the Iba1 + phenotype of reactive microglia in the retina of RP patients, increase the number of microglia and enable microglia to migrate to the deeper retina[86].

#### Anti-inflammatory effects of nanoparticles

There are few experiments in which NPs are used to treat RP. The mechanism involved mainly inhibits the activation of microglia by silencing related genes.

In 2021, Merve Sen et al. used MNPs and magnetic forces to deliver VCP siRNA into retinal explants to target VCP, the therapeutic target of adRP. They demonstrated that reverse magnetoreception of VCP siRNA can effectively silence VCP, which further attenuated Iba1 activation, reduced microglial activation and inhibited associated inflammation. In addition, the inhibitory effect on VCP could also produce a protective function on the optic nerve[49]. Furthermore, TGF-beta receptor (TGFBR) 1 and TGFBR2 are highly expressed in microglia, which also provides the necessary conditions for the anti-inflammatory cytokine TGF-β to inhibit microglial activation[87].

### Age-related macular degeneration

AMD is a chronic retinal degeneration associated with Mps infiltration into the subretinal space, with the impairment of RPE function. Moreover, the interaction between Mps and the RPE is also involved in the pathogenesis of AMD. At the same time, inflammation, neovascularization, and oxidative stress are direct pathological outcomes of AMD[14, 52, 88, 89]. Clinically, medium vitreous warts and retinal pigment changes are early lesions, and neovascularization and atrophy are late lesions. [67] In addition, best dystrophy (BD), or best vitelliform macular dystrophy (BVMD), is another form of macular degeneration that mainly occurs in teenagers and can cause a defect in the central field of vision[90].

#### Inflammatory mechanisms involved in the pathogenic process

RPE cell death is a crucial link in the progression of AMD. Retroactively, abnormal activation of the innate immune system, including the activation of retinal microglia and macrophages, the overexpression of complement, chemokines and inflammatory factors, the accumulation of lipofuscin, and the disruption of lysosomal enzyme activity, can contribute to the progression of AMD. IL-1β induces the assembly of the NLRP3 inflammasome, recruitment of macrophages, and activation of IL-6 and chemokines[84, 91]. Due to the ability of macrophages to resist clearance by RPE cells, subretinal macrophages may contribute to the loss of cone photoreceptor function in advanced AMD[92]. Moreover, more macrophages can be induced to accumulate in the retina when RPE cells secrete more MCP-1[93]. IL-18 is suspected to play a role in retinal degeneration inflammation by inducing IFN-γ. However, because IL-18 can reduce CNV, it shows a protective effect in wet AMD. IL-33 can be produced by Müller cells and RPE cells stimulated by Aβ. IL-33 activates the NF-κB and MAPK inflammatory signaling pathways and induces the production of inflammatory cytokines (including IL-1β, IL-6, IL-8, and TNFα) and chemokines (including C–C motif chemokine ligand 2 (CCL2)) to recruit macrophages[84]. The chronic inflammation caused by these inflammatory factors can increase VEGF and induce neovascularization[94], pathological processes such as Bruch membrane degradation and RPE death. Moreover, complement C5B-9 is also involved in the pathogenic process of choroid atrophy. In addition, oxidative stress significantly promotes RPE cell death and AMD progression. The high metabolism of the RPE leads to the production of more ROS for signal transduction. However, ROS levels are still within the physiological range for RPE cells because RPE cells function in the p62/Keap1/Nrf2 pathway to activate autophagy-induced antioxidant function and neutralize excessive ROS. With age, senescent cells in the body can produce more ROS, and RPE cells become more sensitive to ROS, which ultimately leads to their death[92]. In addition, MtDNA damage is more common in people with AMD, so there may also be a causal link[95].

#### Anti-inflammatory effects of nanoparticles

The mechanism of NPs in the treatment of AMD mainly involves targeting abnormally activated inflammatory cells, inhibiting the excessive production of chemokines, downregulating the release of cytokines, resisting excessive oxidation, and resisting tissue cell apoptosis.

The most direct effect is to target inflammatory cells to affect the expression and secretion of inflammatory cytokines and chemokines. Siva P. Kambhampati et al. used generation 4 hydroxyl-terminated PAMAM dendrimers (D4-OH) to encapsulate TA for nonvitreous administration because D4-OH administered intravenously or orally could penetrate through the damaged blood‒retinal barrier. After intravenous injection of Cy5-labeled dendrimer-triamcinolone acetonide (D-TA) (Cy5-D-TA), they found that Cy5-D-TA targeted activated microglia, Mps, and RP cells, which could crosstalk with neighboring cells to release proinflammatory cytokines and chemokines and proangiogenic factors that promote AMD progression. As expected, the expression of proinflammatory cytokines (TNF-α, IL-1β, IL-6) and inflammatory chemokines (MCP-1, ICAM-1) was significantly inhibited[14].

As mentioned above, the increase in MCP-1, which is produced by RPE cells to induce macrophage recruitment, trigger local inflammation, and increase the secretion of VEGF by Mps, has the potential to cause AMD. Consequently, Zhao-Jiang Du et al. developed a strategy of triple transplantation of RPE cell-MCP-1 antibody-VEGF antibody compounds. They proposed that superparamagnetic iron oxide nanoparticles (SPIONs) can be used to precisely target the compound to the macula using magnetic navigation[93]. Based on the antioxidant and anti-inflammatory properties, including the inhibition of NF-κB and HIF-1α transcription factors, Priyanka Bhatt et al. used PLGA to load RES for the treatment of AMD. Compared with free RES, PLGA-RES could be absorbed by ARPE-19 cells more efficiently and reduce VEGF expression significantly, demonstrating that more RES was effective in playing a therapeutic role. Although not experimentally proven, it was not difficult to speculate that the NF-κB and HIF-1α transcription factors, as one of the anti-VEGF mechanisms of RES, were also significantly downregulated[13].

However, long-term inhibition of VEGF can lead to adverse effects on the retina. Consequently, using therapeutic agents with antioxidant, anti-inflammatory, and antiangiogenic functions is an ideal therapeutic strategy. Nanoceria is considered an alternative therapy because of its ability to eliminate ROS and inhibit apoptosis. To compensate for nanoceria’s low water solubility and poorly controlled release ability, Kai Wang et al. developed an injectable system consisting of natural oligo-chitosan-coated nanoceramics (OCNPs). The results demonstrated that this administration pathway could effectively inhibit the oxidative stress response, inhibit the apoptosis of ARPE-19 cells, and downregulate the expression of the inflammatory factors TNF-α and IL-6, which is essential for preventing the development of early AMD[59].

### Choroidal neovascularization and retinal neovascularization

Retinal and choroid neovascularization are common ocular lesions. In addition to the DR and AMD mentioned above, which lead to retinal and choroid neovascularization, many other ocular diseases also lead to neovascularization[96, 97]. There is a close relationship between inflammation and angiogenesis, which involve common pathways and can be mutually induced[31, 98].

#### Inflammatory mechanisms involved in the pathogenic process

VEGF upregulation and immune cell infiltration synergistically promote CNV production. Various proinflammatory factors, especially IL-6, IL-8, MCP-1, TNF-α and NF-κB, can promote the upregulation of VEGF[99, 100]. VEGF-A activates VEGFR2 to promote neovascularization, while VEGFR1 acts as a VEGF-A decoy to block VEGFR2/VEGF-A-mediated reactions, which are essential in homeostasis. The overexpression of placental growth factor (PLGF) is closely related to the promotion of retinal microaneurysms and angiogenesis. PLGF inhibition increases Akt phosphorylation and inhibits the HIF-1α-VEGF pathway, preventing retinal cell death, capillary degeneration, pericyte loss, and blood‒retinal barrier (BRB) breakdown. It has also been demonstrated that PLGF-induced VEGFR1 activation upregulates the expression of inflammatory factors such as TNF-α and IL-6, which further promotes VEGFR1 expression. In addition, hypoxia can induce the overexpression of inflammatory factors that promote angiogenesis. VEGF-A expression can also be upregulated in astrocytes and Müller glial cells with increased oxygen consumption[100, 101]. VEGF itself or VEGF under PLGF stimulation can induce the migration of macrophages. MMPs (mainly including MMP-2 and MMP-9) produced by infiltrated macrophages can reshape the extracellular matrix, thus promoting the infiltration of inflammatory cells. VEGFR1 also causes macrophages and microglia to secrete proinflammatory and proangiogenic mediators (including CCL2, IL-1β, IL-6, TNF-α, and VEGF-A) in the retina, and this inflammatory microenvironment further promotes macrophage persistence[99]. The polarization of macrophages also affects angiogenesis, with the M2 phenotype promoting angiogenesis and the M1 phenotype inhibiting angiogenesis. In addition, the activation of the RhoA/ROCK signaling pathway plays an essential role in the formation of CNV[102].

#### Anti-inflammatory effects of nanoparticles

The inflammatory mechanisms involved in the treatment of DR by NPs include preventing inflammatory cell infiltration and downregulating the expression of inflammatory cytokines and chemokines by targeting cytokines or related genes directly.

Alper Ozturk et al. found that DS-loaded PLGA NPs increased the penetration capacity of DS into the chorioallantoic membrane (CAM), which enhanced anti-inflammatory and anti-angiogenesis activity at low drug doses[31]. Based on the characteristics of AuNPs that were easily internalized by cells and tissues, Md Imam Uddin et al. constructed AS-VCAM-1 hAuNPs, which contained antisense sequences complementary to VCAM-1 mRNA, to aggregate to AMD-induced neovascularization. These AS-VCAM-1 hAuNPs showed good binding and imaging function to VCAM-1 mRNA. VCAM-1 is an inflammatory marker that can be induced by the proinflammatory cytokine TNF-α. This binding site and binding mode provide an excellent reference for the targeted delivery of drugs[39]. Kunbei Lai et al. then used APRPG, a short peptide that explicitly targets VEGFR-1, to address the low specificity of TP. They used nanoliposome-APRPG-loaded TP for the treatment of CNV. They found that drug uptake by EA.hy96 endothelial cells increased after APRPG peptide modification. The results showed that M2 macrophage infiltration and M2-induced cell proliferation and migration were significantly inhibited in anti-inflammatory cells. The protein levels of VEGF, ICAM-1, and MCP-1 were also significantly downregulated[40].

In addition, exosomes have been shown to reduce macrophage infiltration and inhibit the production of many immunomodulators, such as TGF-β, IL-4, and IL-10, by regulating cells, such as the RPE. However, Yai-Ping Hsiao et al. found exosomes possessed different particle sizes in the aqueous fluid of patients with different types of retinal neovascularization, suggesting that exosomes may be a potential target for treating ocular diseases through anti-inflammatory mechanisms[103].

### Endophthalmitis

Endophthalmitis is a severe intravitreal inflammatory disease, often secondary to other ocular diseases, such as cataracts, and can cause damage to other areas, such as the retina. Endophthalmitis can be caused by bacteria (Gram-positive bacteria such as *Staphylococcus aureus* and *Streptococcus* and Gram-negative bacteria such as *Klebsiella* and *E. coli*) invading the eye, which can secrete pore-forming toxins and cause blindness[104, 105].

#### Inflammatory mechanisms involved in the pathogenic process

Some immune components can be suppressed to inhibit Th1 and NK-cell activation, reduce endotoxin-induced inflammation, and induce macrophages to produce anti-inflammatory cytokines, including alpha-melanocyte-stimulating hormone (α-MSH), vasoactive intestinal peptide (VIP), TGF-β2, prostaglandin E2, etc. After Gram-positive cell infection, these immune components are reactivated. Specific processes include the activation of TLRs (especially TLR4 and TLR2), recruitment of immune cells to infection sites and secretion of a variety of cytokines. In Bacillus endophthalmitis, the activation of TLR4 and TLR2 could promote the signal transduction of the downstream adaptors MyD88 and TRIF and eventually induce the aggregation of polymorphonuclear leukocytes[106]. Among the inflammatory cytokines, IL-8 recruits leukocytes, IFN-γ activates macrophages, IL-2 and IL-6 activate lymphocytes, and growth factors promote tissue repair. Neutrophils directly cause retinal damage, which is facilitated and exacerbated by CXCL1[107].

#### Anti-inflammatory effects of nanoparticles

In the treatment of endophthalmitis, NPs can inhibit the survival of microorganisms, the expression of inflammatory factors and the activation of inflammatory cells by delivering drugs or acting directly.

Yang Ye et al. designed novel AuAgCu2O-bromfenac sodium (AuAgCu2O-BS) NPs based on the antibacterial properties of Ag ions, Ag-based compounds, and Cu ions and the excellent ocular anti-inflammatory properties of bromfenac. The AuAgCu2O-BS NPs eventually destroyed almost all bacteria and suppressed inflammation by downregulating elevated IL-1β and IL-6. Specific mechanisms included methicillin-resistant *Staphylococcus aureus* (MRSA) elimination through mild metal ion release photothermal action and inflammatory response inhibition by sodium bromophenolate[54].

Binapani Mahaling et al. designed PLA core and CHI shell NPs to load AZM and TA based on the hydrophilicity and mucosal adhesion of CHI. The dual-drug delivery system enhanced the anti-inflammatory and antibacterial activity of each drug, which inhibited microglial activation and decreased IL-1β levels[45].

## Other diseases

**Fig. 6:**
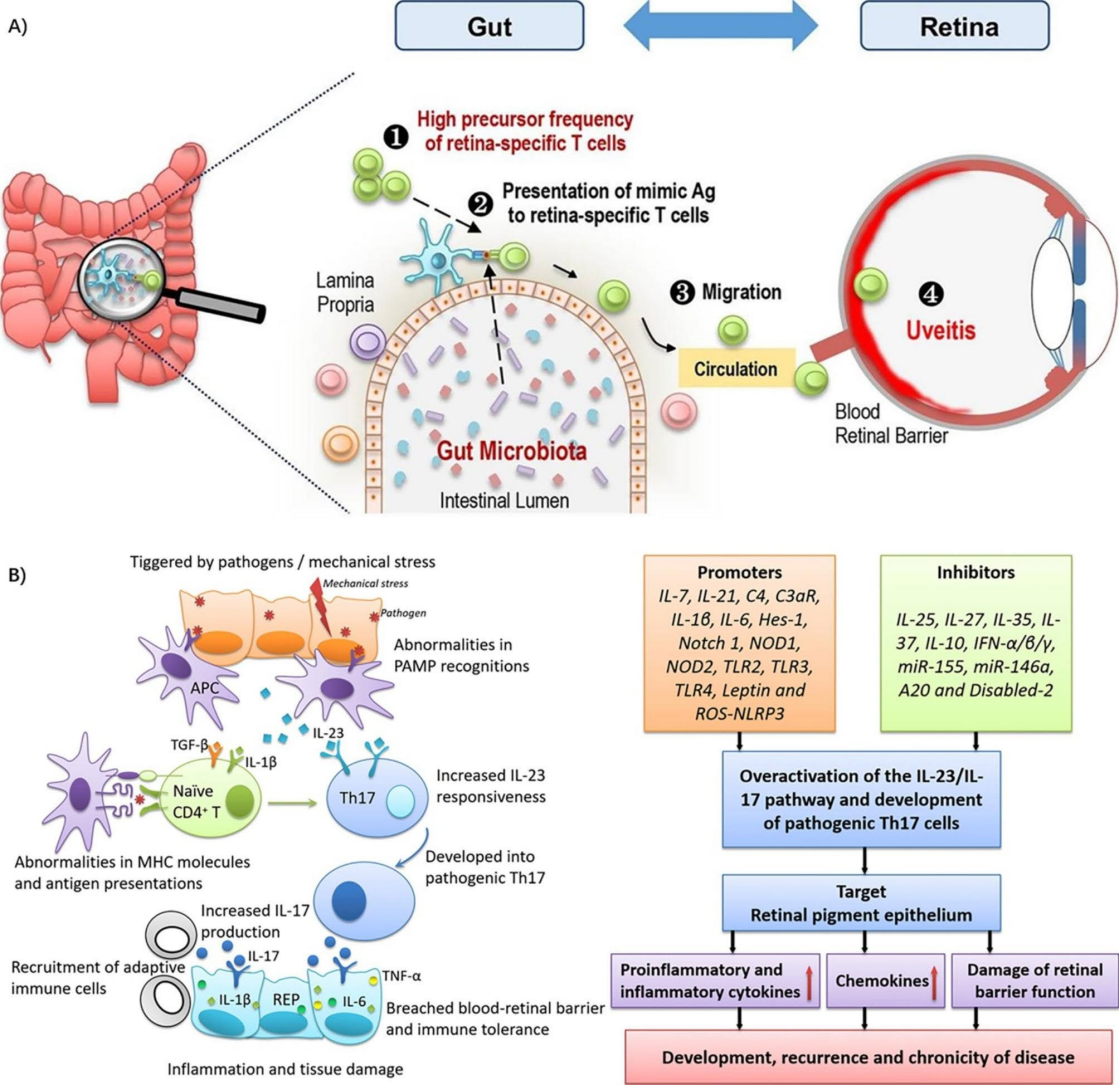
Model diagram of the inflammatory mechanism involved in uveitis and optic nerve injury. (A) Retina-specific autoreactive T cells distributed diffusely in the intestinal tract and associated uveitis. (B) Inflammatory response and IL-23/IL-17 signaling in autoimmune uveitis. Reproduced under the terms of the CC-BY 4.0 license [111]. Copyright 2019, The Authors. Published by Frontiers Media SA. Reproduced with permission [112]. Copyright 2021, Elsevier.

Here, we summarize the inflammatory mechanism involved in the pathogenesis of uveal diseases, glaucoma and visual pathway diseases and the anti-inflammatory mechanism of NPs in the treatment of these diseases (as shown in Fig. 6; Table 3).

**Table 3:** The application of nanoparticles in other diseases

Citation	NPs	Diameter	Function of NPs	Anti-inflammatory mechanisms	Animal models	
[114]	EV-mPEGhexPLA	/	Deliver EV	Decrease IL-2, IL-17, and IFN-γ secretion; increase IL-10 secretion; and increase the frequency of CD4 + CD25 + FoxP3 + Treg cells	B10.RIII mice with EAU	
[115]	Nano-SOD1	44–45 nm	Deliver SOD1	/	Chinchilla rabbits with immunogenic uveitis induced by injecting normal horse serum	
[6]	TA-mPEG-PLGA NPs	40 ~ 100 nm	Deliver TA	Inhibit the expression of IL-17 and IL-10	Lewis rats with EAU induced by interphotoreceptor retinoid binding protein (IRBP) emulsion	
[41]	PLGA-GA_2_-CUR	250 nm	Deliver CUR	/	Beagles with homologous lens protein-induced uveitis	
[33]	TA-SA/PECE	30 nm	Deliver TA-SA	Inhibit the production of NO and TNF-α by activated RAW264.7 macrophages, inhibit IL-6 expression, reduce neutrophil infiltration	Japanese big-eared white rabbits with LPS-induced EIU	
[28]	TA-PLC NPs	120 nm	Deliver TA	Decrease the expression of IL-6	Albino rabbits with endotoxin-induced uveitis	
[29]	cTA-LNA NPs	198.95 ± 12.82 nm	Deliver TA	Reduce TNF-α levels, reduce inflammatory cells, reduce bleeding and congestion	HET-CAM	
[34]	CUR-NPs	161.0 ± 6.0 nm	Load CUR	Downregulate the expression of TNF, IL-1α, IL-6 and MMP-13; inhibit TM cell apoptosis	New Zealand albino rabbits	
[50]	PAMAM-PVL-PEG	34 nm	Deliver dehydroepiandrosterone (DHEA) and NMDA	Inhibit microglia/macroglia activation and oxidative stress	C57BL/6 MICE with NMDA-induced RGC degeneration	
[55]	FK506 loaded nanomicelle and ciliary neurotrophic factor (CNTF)	100–350 nm	Deliver CNTF and FK506	Inhibit microglial activation	Japanese white rabbits with optic nerve damage caused by reverse arterial clamp clamping	

### Uveal diseases

The uvea is the middle layer of the three-layer structure of the eye, including the choroid (vegetative-vascular tissue), ciliary body, and iris[108]. In addition to being classified according to anatomy, uveitis can also be divided into infectious uveitis and noninfectious uveitis according to its etiology. Herpes and toxoplasmosis are the leading causes of uveitis, and both Th1 and Th17 T cells and cytokines such as IL-6 and TNF-α are involved in the pathogenesis of uveitis[108, 109]. Noncommunicable uveitis is generally more common in developed countries. It is an ocular disease that can cause blindness and is often treated with corticosteroids to prevent inflammatory eye complications[110].

#### Inflammatory mechanisms involved in the pathogenic process

As an autoimmune disease, experimental autoimmune uveoretinitis (EAU) is associated with HLA, and different types of uveitis are associated with specific HLA haplotypes, including A29, B27, B51, DR4, and DQ4[111]. The pathogenesis of EAU involves the IL-23/IL-17 pathway, which promotes Th17 amplification. Th17 cells and their secreted IL-2 and IFN-γ also play a significant role. IL-17 may also activate downstream inflammatory cascades by targeting RPE cells, resulting in damage to these cells and the retinal barrier[112]. Damage to the retinal barrier results in the release of retinal autoantigens into the peripheral blood, which activates reactive T cells in the peripheral blood and causes disease[113]. The early stage of EAU is characterized by microglial migration, while the late stage is characterized by macrophage recruitment. In both stages, the IL-1 family of inflammatory factors plays an essential role. For example, IL-33 and IL-1β secreted by neutrophils, macrophages, and DCs were elevated in the inner core of EAU mice. IL-1R contents also show a positive correlation with the severity of EAU by influencing inflammatory cell recruitment to the retina. Interestingly, however, in the EAU model of wild-type mice, the use of IL-33 reduced inflammation, including T cells, IFN-γ, and IL-17[84]. Furthermore, Reiko Horai et al. suggested that T cells activated by the gut microbiome may be pathogenic by targeting retina-specific homologous antigens[111].

#### Anti-inflammatory effects of nanoparticles

The inflammatory mechanisms of NPs in uveitis mainly include inhibiting the excessive secretion of inflammatory factors, recruitment and infiltration of inflammatory cells in aqueous solution, aggregation of other proinflammatory components, and leakage of inflammatory proteins.

Many NPs have shown the ability to reduce the levels of inflammatory cytokines in the anterior chamber, and some even further affect the activation and infiltration of inflammatory cells. For example, MarenKasper et al. used methoxy polyethylene (ethylene glycol)-hexyl-substituted poly(lactic acid) (mPEGhexPLA) to load everolimus (EV) based on its small size and inert surface properties, facilitating drug passage through biological barriers. After therapy, IL-2 production and the amount of Treg cells in the right lymph node were reduced, and IFN-γ production was reduced in splenic cells. However, CD3 + CD4 + cells and Treg cells in the spleen and IL-2, IL-17, and IL-10 secreted by spleen cells were not significantly changed[114]. Hydrophilic mPEG can control the biodegradation rate of PLGA, so Dadong Guo et al. used mPEG-PLGA NPs to load TA to prolong the efficacy of the drugs, which was finally proven to maintain drug release for 45 days. In terms of anti-inflammatory effects, mPEG-PLGA NPs loaded with TA significantly reduced inflammatory cells compared to NPs without drugs. Furthermore, both TA intravitreal injection and TA-loaded mPEG-PLGA NPs showed significant inhibition of IL-10 and IL-17, two cytokines closely associated with uveitis[6].

On this basis, the introduction of coatings with a positive charge on the surface, such as CHI and L-arginine, has been used in some experiments to increase the drug residence time and effectivity. Nirbhavane P et al. designed a TA-loaded NLC with a positive cationic charge provided by stearic amine on the surface. They demonstrated that this TA-NLC inhibited TNF-α more significantly than free TA, potentially benefiting from enhanced drug permeability[29]. Yi Xing et al. used PLGA to load TA and coated it with cationic CHI polymers to enhance the biocompatibility and cell targeting and uptake ability mediated by a positive charge. Albino rabbits with endotoxin-induced uveitis were selected as the experimental animals. It was shown that these NPs inhibited IL-6 overproduction and improved inflammatory symptoms, such as reducing aqueous humor turbidity to reduce blurred vision[28]. In addition, based on previous studies showing that superoxide dismutase 1 (SOD1) treatment could reduce the infiltration of retinal inflammatory cells, Alexander N Vaneev et al. designed double-coated SOD1 nanoparticles (nano-SOD1), which were first combined with a polycation and then coated with the polyanionic block copolymer. In addition to antioxidant effects, nano-SOD1 also produced anti-inflammatory effects, such as decreasing the inflammation score, fibrin clots in the aqueous humor, and the total protein content in the aqueous humor[115].

More interestingly, Jinhai Huang et al. formed TA-SA/PECE by mixing a TA-SA supramolecular hydrogel with a PECE aqueous solution. The results showed that TA-SA/PECE NPs could penetrate the drugs in the form of TA-SA and TA simultaneously, which significantly improved the efficiency of drug delivery. This administration strategy significantly inhibited the production of NO and TNF-α by activating RAW264.7 Mps, inhibited IL-6 expression, reduced neutrophil infiltration, and ultimately reduced the symptoms of anterior chamber inflammation[33].

The anti-inflammatory effect of NPs can also be shown simply as a decrease in aqueous humor turbidity and the inflammation score, which are associated with a decrease in inflammatory cells and the inflammatory substances they produce in aqueous humor. For instance, the PLGA-GA2-CUR-NPs designed by R Ganugula et al. successfully downregulated inflammatory parameters, including nanaqueous flares and conjunctival parameters, up to 6 h after use[41]. M L Formica et al. used NLCs to load TA. In the endotoxin-induced uveitis (EIU) model, TA-NLCs further reduced the ability of other inflammatory cells to secrete other inflammatory factors by influencing the secretion of IL-6. In addition, because protein leakage caused by inflammation could cause aqueous humor turbidity, they found that TA-NLCs could reduce aqueous humor turbidity and improve visual function[7]. Xinxin Yu et al. designed a Dex peptide conjugated by a cleavable ester bond (Dex-SA-FFFE), which can spontaneously form NPs in an aqueous solution. Since there is no drug carrier, safety problems related to the carrier can be avoided. Based on the diversity of amino acids, the NPs could fine-tune the amphiphilicity of the drugs, thus maintaining high permeability in different environments. In anti-inflammatory experiments, Dex-SA-FFFE significantly inhibited the production of IL-6 and TNF-α. At the same time, Dex-SA-FFFE could also reduce the number of inflammatory cells in the aqueous humor, which alleviated the symptoms of uveitis[116].

### Glaucoma

Glaucoma is known to be the second leading cause of blindness worldwide[117, 118]. Cellular senescence, mitochondrial dysfunction, oxidative stress, and neuroinflammation are all involved in the process of glaucoma. After the accumulation of inflammatory compounds exceeds the compensatory capacity, intraocular pressure (IOP) may increase, leading to a loss of TM cells and retinal ganglion cells (RGCs) or the worsening of optic nerve hypoplasia (ONH). Thus, it is necessary to use anti-inflammatory drugs in the early stage of the disease[119]. Glaucoma has three forms: open-angle glaucoma, angle-closure glaucoma and secondary glaucoma. Open-angle glaucoma is divided into high-pressure glaucoma and low-pressure glaucoma, known as primary open-angle glaucoma and normal intraocular pressure glaucoma, respectively.

#### Inflammatory mechanisms involved in the pathogenic process

The earliest inflammatory pathways involved are TLR-activated MHC II upregulation and cytokine production to stimulate T-cell proliferation. Subsequently, the NF-κB pathway is activated to further increase IL-1 family expression and promote downstream microglia and astrocyte cascades[120]. Activated microglia can be divided into M1 and M2 microglia. M1 mainly secretes proinflammatory cytokines (IL-1β, IL-12 and TNF-α) to induce neurotoxicity, while M2 produces anti-inflammatory mediators (IL-10 and TGF-β) and IGF-1 to promote tissue repair[119]. In addition, high IOP itself and increased levels of inflammatory bodies (such as Nlrp3, Casp1, ASC and IL-1β) also contribute to the progression of inflammation[84].

#### Anti-inflammatory effects of nanoparticles

The use of immune NPs in glaucoma is not common; however, this does not mean that NPs are not suitable for glaucoma treatment. Taking angle-closure glaucoma as an example, the traditional treatment of glaucoma is through autonomic nervous system modulating drugs such as pilocarpine or through procedures such as peripheral iridoplasty and peripheral iridotomy. Therefore, NPs play a role in treating glaucoma mostly by achieving sustained drug release, improving the targeting ability and reducing the systemic absorption of the injected drug, but not by involving inflammatory mechanisms. Among the few experiments utilizing anti-inflammatory mechanisms, reducing the expression levels of inflammatory factors and oxidative stress levels are the main mechanisms.

CUR has been used in the treatment of many ocular diseases due to its ability to block downstream pathways of TNFα, such as NF-κB. As mentioned above, simultaneous delivery of CUR-NPs and latanoprost successfully prolonged the effective duration of a single administration and reduced the expression of TNF, IL-1α, IL-6 and MMP-13 in TM cells and the production of ROS in mitochondria[34].

### Visual pathway diseases

TON refers to a pathological condition of the optic nerve caused by external direct or indirect damage, which can be caused by the destruction of RGC axon fibers or cell death.

#### Inflammatory mechanisms involved in the pathogenic process

In the model of optic nerve injury, RGC survival is facilitated by lymphocyte infiltration at the injury site, which is associated with retinal microglia and macrophage infiltration. The infiltration of these two cell types can promote the formation of CD4 + CD25 + T cells by secreting cytokines, which further promotes the survival of RGCs. Moreover, microglia can also promote Wallerian degeneration and axon regeneration. Nevertheless, when microglia and macrophages are depleted, neuron loss is inevitable[121, 122]. At the same time, microglia can also produce cytokines, chemokines and ROS, which have neurotoxic effects on RGCs. Astrocytes can form myelin fragments and glial scars that inhibit the regeneration of RGC axons, resulting in unsatisfactory visual function recovery of TON. Nevertheless, normal activation of microglia and influx of bone marrow cells are beneficial for RGC axon regeneration and nerve repair[122].

#### Anti-inflammatory effects of nanoparticles

In the model of optic nerve injury, the therapeutic mechanism of immune NPs involves the inhibition of inflammatory cell activation, promotion of damaged nerve regeneration through some inflammatory signaling pathways, and inhibition of excessive oxidative stress levels.

The regeneration ability of damaged optic nerve axons is limited. Currently, no axon connection method can effectively promote optic nerve regeneration or improve function after traumatic optic neuropathy. Since RGC death can occur within a short period (5–14 days) after axon injury, ensuring the potential for RGC axon regeneration is key to optic nerve repair. Ciliary neurotrophic factor (CNTF) has been shown to promote neurite growth in RGCs by activating the Janus kinase (JAK)/signal transducer and activator of transcription (STAT) 3-, phosphoinositide 3-kinase (PI3K)/V-akt murine thymoma viral oncogene homolog (AKT)-, and MAPK/ERK signaling pathways, and tacrolimus (FK506) can inhibit the overactivation of immune responses leading to neurotoxicity. Dongmei Wang et al. designed a CHI thermosensitive hydrogel loaded with FK506 micelles and CNTF to promote the repair of optic nerve injury. Amphiphilic polymer micelles enabled successful encapsulation of hydrophobic FK506 into hydrogels. They used Japanese white rabbits with optic nerve damage caused by reverse arterial clamp as animal models and found that there were fewer Iba-1-positive cells after treatment, indicating that microglial activation after TON was successfully suppressed[55]. Dehydroepiandrosterone (DHEA) is a Sigma-1 receptor (S1R) agonist that is considered a potential intervention to prevent RGC death. Lei Zhao et al. combined NPs with cholera toxin binding B domain (CTB) targeting RGCs and finally designed a DHEA-loaded CTB-unimNPS. It effectively protected RGCs from N-methyl-D-aspartic acid (NMDA)-induced retinal cell death, the mechanisms of which may include the inhibition of microglia/macroglia activation and inhibition of oxidative stress[50]. In view of the harm caused by excessive ROS produced in the optic nerve compression (ONC) process, Xiaotong Lou et al. used PDA NPs to deliver brimonidine to the ONC model, among which PDA was characterized by ROS scavenging due to its rich phenolic groups, and brimonidine played a neuroprotective role by regulating the activity of NMDA. The results showed that this treatment regimen had a long-term protective effect on RGC loss and visual impairment by reducing ROS levels in the retina, inhibiting macrophage polarization, and inhibiting microglial activation after ONC[123].

## Conclusion and further perspectives

Various side effects of conventional medications, including weak drug permeability, a fast drug metabolism rate, and low patient compliance, limit their further popularization in the treatment of ocular diseases. As an emerging substance to improve drug properties or even directly replace drugs, NPs have become a new research hotspot. In the past decade, research in nanomedicine has shown a tendency of significant growth, which implies excellent potential for the gradual application of NPs in clinical practice.

This review summarizes the studies using NPs to treat different ocular diseases through anti-inflammatory mechanisms in the last five years. In ocular diseases, multiple inflammatory pathways are activated (including the TLR4, NF-κB, LFA/ICAM-1, JNK and P38 MAPK pathways), which further promote the secretion of proinflammatory cytokines. Elevated inflammatory factors further stimulate the recruitment, activation and even differentiation of immune cells to a sensitization state, which will cause damage to the lesion site. Chemokines such as CXCL and CXCR also play an essential role. In addition, autophagy, the activation of the complement system, oxidative stress, the regulation of TLR and the activation of microglia are closely related to the severity of disease by influencing the level of inflammatory factors. As we have emphasized in the previous discussion, traditional anti-inflammatory drugs are limited by the physiological barriers of the eye, the limited solubility of drugs, invasive drug delivery routes, etc. Hence, it is urgent to design a drug carrier to achieve efficient drug delivery or even develop new types of substances to directly play the anti-inflammatory role of traditional drugs. There is no doubt that NPs deliver on both expectations. On the one hand, NPs are designed as carriers of anti-inflammatory drugs and other biological agents to enhance the permeability of drugs across ocular physiological barriers, improve the biocompatibility and stability of drugs, prolong the drug release time, reduce the excessive loss of drugs, realize the inflammation-response release of drugs, regulate the precise targeting ability of drugs, achieve non-invasive drug delivery and reduce the side effects of drugs. On the other hand, some nanomaterials, including naturally sourced exosomes, metallic AuNPs and nonmetallic SeNPs, can function independently to block related inflammatory signaling pathways without any drugs. In addition, hybrid nanosystems with multiple materials have also been designed to realize various functions simultaneously. The inflammatory regulation that these NPs participate in mainly includes modulating cytokine production, modulating immune cell activation and differentiation, inhibiting immune cell infiltration, alleviating oxidative stress, and blocking the excretion of other inflammation-related molecules. The wide application potential of NPs is closely related to their variable physical and chemical properties, such as their size, shape, surface morphology, composition, structure, and density. The general advantages of NP ocular applications include increasing drug permeability, prolonging drug release, and enhancing targeting capacity. However, the requirements and emphases when treating specific diseases are different. For example, drugs can easily reach the cornea through eye drops or subconjunctival administration, and the main role of NPs in corneal diseases is to improve the residence time of drugs in the cornea and delay drug release. Conjunctival lesions have relatively lower requirements for advanced drug delivery systems. Vaccine carriers are unique applications of NPs in conjunctival diseases. For lens-related diseases, such as cataracts, the drug needs to penetrate through the cornea and spread through the aqueous humor after surface administration, which can be achieved by adding coatings, groups, and charges to the surface of NPs. For NPs targeting the lens, it is necessary to take into account whether they can cause lens opacity. In addition, studies have reported that 5-fluorouracil can prevent the posterior capsule opacities induced by lens replacement surgery, and NP encapsulation can reduce the incidence of 5-fluorouracil-induced endophthalmitis. NPs targeting the retina need to penetrate through the BRB, so there are high requirements for sizes and surface charges. For example, 20 nm but not 100 nm systemic gold NPs can penetrate through the BRB. Recently, comprehensive research on NPs in the treatment of ocular diseases has shown a significant increasing annual trend, and NPs modified by active targeting ligands and NPs with stimulus-response functions have become the latest research hotspots. Typical components of therapeutic NPs are polymers or lipids. However, NPs with similar compositions can play various roles through different modifications. In addition, while drug delivery to improve the performance and efficacy of drugs is still the most common application of NPs, an increasing amount of research in other applications has been carried out as more therapeutic properties of NPs have been elucidated.

Clinical trials of NPs for ocular diseases have been underway since 2013, involving many types of NPs and many types of diseases, but mostly in patients with DED and glaucoma. Based on the effect of the anti-inflammatory agent cyclosporine A (CsA) in DED treatment, Min-Ji Kang et al. conducted a clinical trial on a cyclosporine A 0.05% nanoemulsion in 2020. In addition to the safety of the nanoemulsion, they also demonstrated that they could improve bioavailability and ultimately significantly downregulate the mRNA expression of IL-6 and MMP-9[124]. Along with experiments related to the anti-inflammation mechanism, Yulian Pang et al. prepared gold nanoparticle (GNR) @ palladium (Pd) hydrogel eye patches for patients with DED. The eye patch made of the NPs was proven to spontaneously heat up under visible light to promote the LG to secrete tears[125]. In 2014, Fengzhen Wang et al. successfully used low-molecular-weight CHI-SLN-methazolamide (MTZ) to reduce IOP in 126 glaucoma patients. Moreover, DexNP eye drops showed efficacy in reducing macular thickness and improving vision in patients with DME in the clinical trial of Akihiro Ohira et al. in 2015[126]. Then, in 2016, Alaa H Salama et al. prepared PLGA NPs with surfaces modified by stearic amine and CHI HCl using a thin-film hydro method, which showed good mucosal adhesion and bioavailability[127]. In 2022, Yu Ma et al. used thermal nanozirconia and noticeable bactericidal nanotitanium dioxide closely combined with optical fiber materials to treat pseudopodia. They demonstrated that the nanosystem had an excellent eye relaxation function. It could reduce lens thickness, IOP, and eye fatigue[128]. In these clinical trials using different types of NPs for different diseases, NPs have achieved ideal therapeutic goals and shown a bright future.

Despite numerous breakthroughs in nanotherapeutic strategies for ocular diseases, there are still many challenges ahead for their clinical translation, which provide the following ideas and directions for future research:

1. Slight changes in the physical and chemical properties of NPs can dramatically affect the biological properties of NPs, but the corresponding relationship between them is unclear. Consequently, the characteristics of NPs (including their chemical compositions, sizes, types and drug loading doses, coatings, zeta potential, and dispersion in solution) need to be further studied in more detail.
2. There are differences in ocular structure among different species. Mice, for example, have a thicker retina and a smaller vitreous volume than humans. The limited uses of horses, pigs, dogs, and cats in ophthalmic experiments are mainly due to animal management problems and animal aggression. As a result, the transformation from animal experiments to clinical trials faces the problems of low accuracy and poor analogy, which adds additional obstacles to clinical translation. Therefore, it is imperative to search for animal models that are more consistent with human beings and to deduce a formula to simulate the condition of human eyeballs. Rabbits, which share more anatomical and biochemical characteristics with humans and are more easily obtained and handled, are an alternative to rodents. The eyeball size, optical systems, biomechanical features, and conjunctival cavity volume of rabbits and humans are relatively more similar. Various species of rabbits have been used to induce a variety of disease models, including autoimmune lacrimal gland inflammation[37], optic nerve damage[55], uveitis[28] [115], etc. These rabbit animal models have been used in corneal transplantation, laser refractive surgery, cataract extraction, intraocular lens implantation, glaucoma shunt implantation and other experimental operations. However, relevant research is not in depth, and the advantages and disadvantages of different animal models still need to be further studied.
3. The hydrogel system has served as a nanodrug carrier to reduce drug loss, but its limited volume load constricts its drug loading capacity, especially for large molecules such as proteins. Hence, more effective drug delivery strategies, such as achieving effective adhesion ability of NPs by modifying their surfaces, need to be developed.
4. More preclinical data are needed to demonstrate the safety of NPs before their clinical application. As exogenous substances, the cellular neurotoxicity and immunogenicity of nanomaterials need to be considered. In addition, many cases of intravitreal drug administration-induced endophthalmitis have been reported, so the occurrence of inflammation needs to be closely monitored after intraocular drug administration. NPs, substances with excellent corneal penetration due to their appropriate sizes, possess a potential therapeutic effect on posterior ophthalmic diseases. During the application process, intraocular inflammatory events also need to be prevented.
5. Although NPs with targeted properties are being designed, successful uptake and utilization by the posterior segment of the eyes remain relatively tricky. More research is needed to overcome the ocular barrier and target posterior segment diseases precisely.

As mentioned above, nanotherapy is an emerging technology that is not yet mature for clinical application in the treatment of ocular diseases. The large experimental investment in the field of NPs is ultimately aimed at clinical application. There are differences between animal and human eyes, from their volume to their specific structure. For example, guinea pigs, which lag blood vessels in their retina, cannot reflect the effect of retinal vascular inflammation, which means that experiments with guinea pigs are limited. Although there have been many relevant experimental research results, the clinical conversion rate is still limited. To achieve clinical translation, it is necessary to apply the parameters obtained from experiments on animals to the human body according to certain formulas, which means there are high requirements for appropriate animal models. Therefore, among the several future research directions mentioned above, we believe that searching for animal models whose physiological structure is more consistent with human physiological and pathological conditions is the most urgent need. In addition, further effectiveness and toxicity evaluation is needed before NP translation from animal experiments to clinical practice, which we think is the most basic and important step for the final implementation of practical application. Of course, as the core of nanotechnology research, the most fundamental and important work in future research is to design NPs with better performance as much as possible, including greater bioavailability, stronger targeting, controllable drug effects, and less biotoxicity. Therefore, NPs are not yet replacements for traditional ocular drugs. All these factors limit the wide application of NPs in clinical practice. However, there is no doubt that NP-based therapeutic strategies are valuable research fields to improve the therapeutic effect and reduce the adverse consequences of conventional treatment. With the continuous development of this technology and the comprehensive evaluation of its safety and efficacy, the challenges mentioned above will gradually be addressed. We believe that nanotechnology is very promising for replacing traditional ophthalmic drugs, which deserves more research.

## Data Availability

Not applicable.

## List of abbreviations

AAPBA: acrylamidophenylboronic acid
AC: acute conjunctivitis
ADSCs: adipose-derived mesenchymal stem cells
adRP: autosomal-dominant retinitis pigmentosa
ADSC-Exos: exosomes derived from adipose-derived stem cells
AGA: acrylamido glucopyranose
AGEs: advanced glycation end-products
AKC: atopic keratoconjunctivitis
AKT: V-akt murine thymoma viral oncogene homolog
AMD: age-related macular degeneration
APRE-19: a human RPE cell line
APRPG: ALA-Pro-ARg-Pro-Gly
AS: antisense sequence
ATG: autophagy-related
AuAgCu2O-BS: AuAgCu2O-bromfenac sodium
AuNPs: gold NPs
AZM: azithromycin
BD: best dystrophy
BRB: blood‒retinal barrier
BV-2: mouse microglial cell line
CALT: conjunctiva-associated lymphoid tissue
CAM: chorioallantoic membrane
CASP1: caspase 1
CCL2: C–C motif chemokine ligand 2
CHI: chitosan
CNTF: ciliary neurotrophic factor
CNV: choroidal neovascularization
COX-2: cycloxygenase-2
CsA: cyclosporine A
cSAPs: colloidal self-assembled patterns
CTB: cholera toxin binding B domain
CUR: curcumin
CXCL: chemokine (C-X-C motif) ligand
CXCR: chemokine receptor
Cy5-D-TA: Cy5 labeled dendrimer-triamcinolone acetonide
D4-OH: hydroxyl-terminated PAMAM dendrimers
DAF: decay accelerating factor
DCs: dendritic cells
D-Dex: dexamethasone conjugate
DED: dry eye disease
Dex: dexamethasone
Dexp: dexamethasone sodium phosphate
Dex-SA-FFFE: Dex peptide conjugated by a cleavable ester bond
DME: diabetic macular edema
DS: diclofenac sodium
D-TA: dendrimer-triamcinolone acetonide
DXI: dexibuprofen
EAU: experimental autoimmune uveoretinitis
EB: Evans blue
EIU: endotoxin-induced uveitis
ELP: elastin-like polypeptide
EV: everolimus
FKBP-V48: 48 ELP repeats with Xaa = Val
FSI: 48 ELP repeats with Xaa = Ser and 48 ELP repeats with Xaa = Ile
GA: gambogic acid
GC: glucocorticoids
GCSF: granulocyte colony-stimulating factor
Gd.GC: GC combined gD
GM-CSF: granulocyte-macrophage colony-stimulating factor
GNRs: gold nanoparticles
HCE: human corneal epithelium
HCEs: human corneal epithelial cells
HIF: hypoxia-inducible factor
HLA: human leukocyte antigen
HmA: His 6-metal assembly
HSH: homogenization
HSH/US: homogenization and ultrasonic treatment combination
HSK: herpes simplex virus keratitis
HVEM: herpes virus entry mediator
HYPX: hypoxia
ICAM-1: intercellular adhesion molecule-1
IFI: interferon-inducible protein
IFN: interferon
IL: interleukin
IMP: immune modification particles
IOP: intraocular pressure
ISG: in situ gel
JAK: Janus kinase
JNK: c-Jun N-terminal kinase
KET: ketoconazole
LFA: lymphocyte function-associated antigen
LG: lacrimal gland
LPS: lipopolysaccharide
MAPK: mitogen-activated protein kinase
MARCO: macrophage receptor with collagen structure
MCP: monocyte chemotactic protein
MHC: major histocompatibility complex
MIP: macrophage inflammatory protein
MMP: matrix metalloproteinase
MNPs: magnetic NPs
MOF: metal-organic framework
MOMP: membrane protein
mPEG: methoxy polyethylene glycol
Mps: macrophages
MRSA: methicillin-resistant Staphylococcus aureus
mTOR: mechanistic target of the rapamycin
MTZ: methazolamide
MZL: mizolastine
nano-SOD1: SOD1 nanoparticles
NF-κB: nuclear factor kappa-B
NK: natural killer
NLC: nanostructured lipid carriers
NLRP3: NOD-, LRR- and pyrin domain-containing 3
NMDA: N-methyl-D-aspartic acid
NO: nitric oxide
NPs: nanoparticles
OCCNP: oligo-chitosan coated nanoceramics
ONC: optic nerve compression
ONH: optic nerve hypoplasia
PCL: polycaprolactone
Pd: palladium
PDA: polydopamine
PEA: palmitoylethanolamide
PECE: poly (ethylene glycol) - poly (ε-caprolactone)-poly (ethylene glycol)
PEG: poly ethylene glycol
PF68: Pluronic® F-68
PGE2: prostaglandin E2
PI3K: phosphoinositide 3-kinase
PLA: poly lactic acid
PLA-PEG: poly lactic acid–copoly ethylene glycol
PLGA: poly (lactide-co-glycolide acid)
PLH: poly(L-histidine)
Rapa: rapamycin
RES: resveratrol
RGC: retinal ganglion cell
ROS: reactive oxygen species
RP: retinitis pigmentosa
RPE: retinal pigment epithelium
SeNPs: selenium NPs
SG: salivary gland
siRNA: small interfering RNA
SjS: Sjogren’s syndrome
SLN: solid lipid nanoparticles
SOD1: superoxide dismutase 1
Solutol® HS15: 15-hydroxy stearate
SPIONs: superparamagnetic iron oxide nanoparticles
STAT: signal transducer and activator of transcription
TA: triamcinolone acetonide
TAC: tacrolimus
TA-SA: succinated triamcinolone acetonide
Tem: Tempo
TF: tear film
TfR: transferrin receptor
TGF: transforming growth factor
Th: T helper
TLR: toll-like receptor
TM: trabecular meshwork
TNF: tumor necrosis factor
TP: triptolide
Tra: tranilast
TRPM2: transient receptor potential melastatin 2
US: ultrasonic
UV: ultraviolet light
UV-Ct: UV-inactivated chlamydia trachomatis
VCAM: vascular cell adhesion molecule
VCP: valosin-containing protein
VEGF: vascular endothelial growth factor
VEGFR: vascular endothelial growth factor receptor
VIP: vasoactive intestinal peptide
VKC: vernal keratoconjunctivitis
α-MSH: alpha-melanocyte-stimulating hormone
